# Pleiotropic roles of Ras GTPases in the nematode-trapping fungus *Arthrobotrys oligospora* identified through multi-omics analyses

**DOI:** 10.1016/j.isci.2021.102820

**Published:** 2021-07-08

**Authors:** Le Yang, Xuemei Li, Meihua Xie, Na Bai, Jiangliu Yang, Kexin Jiang, Ke-Qin Zhang, Jinkui Yang

**Affiliations:** 1 State Key Laboratory for Conservation and Utilization of Bio-Resources in Yunnan, and Key Laboratory for Southwest Microbial Diversity of the Ministry of Education, Yunnan University, Kunming 650032, China; 2 School of Life Science, Yunnan University, Kunming 650032, China

**Keywords:** Molecular biology, Mycology, Transcriptomics

## Abstract

The nematode-trapping fungi are ideal agents for controlling pathogenic nematodes. *Arthrobotrys oligospora* is a representative species of the same, producing traps for nematode predation. Here, three orthologous Ras GTPases (Ras2, Ras3, and Rheb) were characterized in *A. oligospora*. Our results indicate that they play pleiotropic roles in regulating the mycelial growth, conidiation, stress resistance, and pathogenicity of *A. oligospora.* Furthermore, deletion of *Aoras2* and *Aorheb* significantly affected the mitochondrial activity, reactive oxygen species levels, lipid storage, and autophagy. Transcriptome analyses of Δ*Aoras2* mutant revealed that many repressed genes were associated with signal transduction, energy production, and carbohydrate transport and metabolism. Moreover, metabolic profile analyses showed that AoRas2 and AoRheb affect the biosynthesis of secondary metabolites in *A. oligospora*. Collectively, these findings provide an in-depth insight into the essential roles of Ras GTPases in vegetative growth, development, and pathogenicity and highlight their importance in the lifestyle switch of the nematode-trapping fungi.

## Highlights

• Ras GTPases play a multifunctional role in the lifestyle switch of *A. oligospora*
• Ras GTPases affect multiple cellular processes, including mitochondrial activity
• AoRas2 plays a key role in regulating global gene expression and nematode predation
• AoRas2 and AoRheb significantly affect the biosynthesis of secondary metabolites

## Graphical abstract

**Figure:**
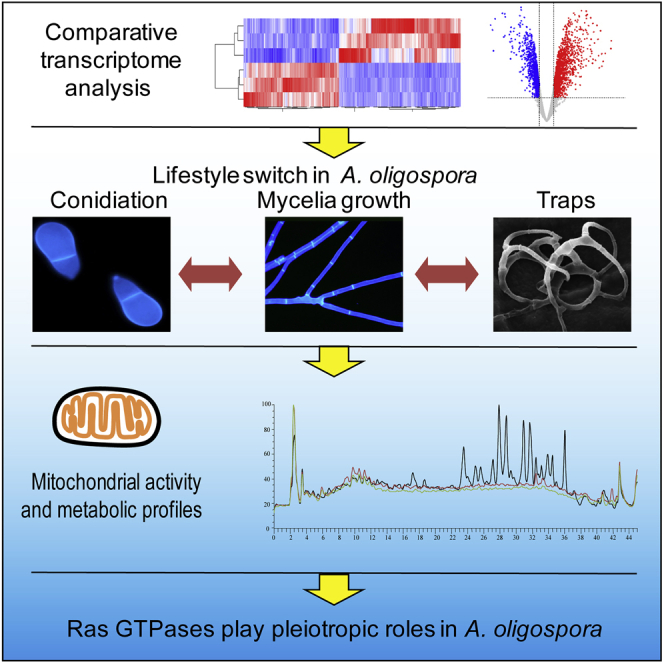

## Introduction

Nematophagous fungi are an important group of soil microorganisms that inhibit the population of the plant and animal parasitic nematodes (Su et al., 2017). The nematode-trapping (NT) fungi, which are the main group of nematophagous fungi, are capable of developing specific trapping structures (traps), such as adhesive networks, adhesive knobs, and constricting rings, to capture nematodes and extract nutrients from them (Li et al., 2005). The trap formation is an important indicator for NT fungi to switch from a saprophytic to a predacious lifestyle (Yang et al., 2011; Ji et al., 2020). Recently, several NT fungi producing different kinds of traps have been sequenced, such as *Arthrobotrys oligospora* (Yang et al., 2011, 2020), *Monacrosporium haptotylum* (syn. *Dactylellina haptotyla*) (Meerupati et al., 2013), *Drechslerella stenobrocha* (Liu et al., 2014), and *Duddingtonia flagrans* (Youssar et al., 2019). These studies provided a broad basis for investigating the mechanisms regulating hyphal growth and development, cell differentiation, and pathogenicity in NT fungi.

The Ras superfamily consists of membrane-associated proteins that display GTPase activity and orchestrate multiple cellular processes (Arkowitz and Bassilana, 2015; Mitin et al., 2005). Ras GTPases are a family of functionally conserved proteins that cycle between GTP-bound (active) and GDP-bound (inactive) conformations and affect the mitogen-activated protein kinase (MAPK) and/or cyclic adenosine monophosphate (cAMP)-protein kinase A (PKA) signaling pathways for the mediation of diverse cellular events in response to external stimuli (Mitin et al., 2005). The fungal Ras GTPase family includes Ras1, Ras2, Ras3, and Ras homolog enriched in brain (Rheb) (Arkowitz and Bassilana, 2015). Ras1 is required for maintaining viability of various filamentous fungi, such as *Aspergillus nidulans*, *Penicillium marneffei*, *Magnaporthe grisea*, and *Fusarium graminearum* (Bluhm et al., 2007; Boyce et al., 2005; Park et al., 2006; Som and Kolaparthi, 1994). Ras2 is dispensable for survival but indispensable for growth, nutrient starvation, cAMP production, cell wall synthesis, conidial formation, and virulence (Bluhm et al., 2007; Kanauchi et al., 1997; Mosch et al., 1999). Ras3 is highly homologous to Ras2 and has a partially overlapping function in radial growth, asexual development, and conidial germination (Guan et al., 2015; Roze et al., 1999). Rheb plays an essential role in controlling growth, cell cycle, and nitrogen starvation by regulating the expression of target of rapamycin (TOR) complex 1 (Aspuria and Tamanoi, 2004, 2008; Bar-Peled and Sabatini, 2014; Wang et al., 2015). In summary, Ras GTPases are functionally versatile proteins; therefore, understanding their common and unique roles in NT fungi is essential for exploring genetic strategies to improve fungal candidate strains and to develop more efficient and persistent nematicides.

*Arthrobotrys oligospora* is one of the best-studied NT fungi as it captures nematodes using adhesive networks (Su et al., 2017; Yang et al., 2020). Its extracellular proteases play an important role in the infection of nematodes (Åhman et al., 2002; Tunlid et al., 1994; Yang et al., 2013). Recently, several proteins involved in signaling pathways are shown to regulate the growth, development, and pathogenicity in *A. oligospora*, such as MAPKs Slt2 (Zhen et al., 2018), Fus3 (Chen et al., 2021), and Bck1 (Xie et al., 2021); Rab GTPase Rab-7A (Yang et al., 2018); G-protein β subunit (Yang et al., 2020); and regulators of G protein signaling (Ma et al., 2021). However, the role of Ras GTPases in *A. oligospora* and other NT fungi remains unclear. In this study, orthologs of three Ras GTPases (Ras2, Ras3, and Rheb) were retrieved from *A. oligospora* and further characterized through gene disruption and multiple phenotype analyses. In addition, the transcriptome-wide gene expression analysis was performed to explore the regulatory role of AoRas2 in the vegetative growth and trap formation. Moreover, metabolic profiles of the wild-type (WT) and mutant strains (Δ*AoRas2* and Δ*AoRheb*) were compared.

## Results

### Sequences and phylogenetic analyses of Ras GTPases (AoRas2, AoRas3, and AoRheb)

Three Ras GTPases, namely, AoRas2, AoRas3, and AoRheb, were retrieved from *A. oligospora* using the homologous sequences of *Neurospora crassa* and *Saccharomyces cerevisiae* as queries. The partial sequence properties of these three GTPases are summarized in Table 1. The three GTPases share a conserved p-loop that contains a nucleoside triphosphate hydrolase (IPR027417) and a small GTP-binding protein domain (IPR005225). The phylogenetic tree of small GTPases from diverse filamentous fungi was constructed based on their amino acid sequences, and the orthologs of Ras2, Ras3, and Rheb were separated into three clades (Figure S1).

**Table 1:** Partial sequence properties of three Ras GTPases in *A. oligospora*

Gene	Open reading frame (bp)	Introns	Amino acid residues	Isoelectric point	Molecular weight (kDa)	G-box motifs^a^	CAAX motif^a^
*Aoras2*	778	1	232	8.76	26.17	G1 to G5	CCIL
*Aoras3*	799	3	208	6.15	23.19	G2 to G5	CCTI
*Aorheb*	789	2	185	6.78	20.61	G1 to G5	CVIM

a Conserved motifs are marked in Figure S1A.

### AoRas2, AoRas3, and AoRheb regulate mycelial growth and cell morphology

**Figure 1:**
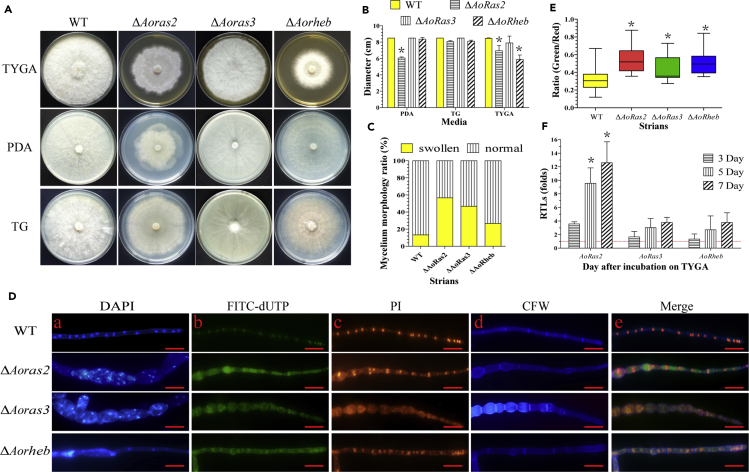
Comparison of colony morphology, mycelial growth, DNA damage, and cell apoptosis in the wild-type (WT) and mutant strains (Δ*Aoras2,* Δ*Aoras3,* and Δ*Aorheb*) of *A. oligospora* (A) Colonies from the WT and mutant strains were cultured on PDA, TG, and TYGA plates for 7 days at 28°C. (B) Colony diameters of the WT and mutant strains cultured on different media for 7 days. Error bars: Data are represented as mean ± SD. The asterisk indicates a significant difference between the mutants and the WT strain (n = 3 for the WT strain, n = 9 for each mutant strain; Tukey's HSD, p < 0.05). (C) The proportions of deformed hyphal cells of the WT and mutant strains cultured on the PDA medium for 7 days. (D) Mycelial morphology and TUNEL assay. The fungi were cultured on the PDA medium for 7 days, and the mycelia were then stained with 4ʹ,6-diamidino-2-phenylindole (DAPI), followed by the TUNEL analysis. a. The mycelia were stained with DAPI. b. Free DNA of the hyphae was re-stained with FITC-dUTP. c. The nuclei of the hyphae were stained with propidium iodide (PI). d. The mycelia were re-stained with Calcoflour White (CFW). e. The merged picture of (b)–(d). Samples were examined under a confocal laser scanning microscope. Scale bar, 10 μm. (E) Analysis of DNA fragmentation and cell apoptosis in hyphal cells. The ratio of green to red fluorescence intensity was determined for at least 30 fields observed under a microscope, and the horizontal bars depict the median. The asterisk indicates a significant difference between the mutants and the WT strain (Tukey's HSD, p < 0.05). (F) Relative transcription levels (RTLs) of *Aoras2*, *Aoras3*, and *Aorheb* genes in the WT strain at different time points. The red line indicates the standard (which has an RTL of 1) for the statistical analysis of the RTL of each gene in a deletion mutant compared with that in the WT strain under a given condition. Error bars: Data are represented as mean ± SD. An asterisk indicates significant difference in the expression of *Aoras2*, *Aoras3*, and *Aorheb* genes in the WT strain at days 3, 5, and 7 days compared with that at day 2 (n = 3 for each gene; Tukey's HSD, p < 0.05).

Three mutants of each Ras GTPase gene (*Aoras2*, *Aoras3*, and *Aorheb*) were generated as described in the transparent methods section and identified by PCR and Southern blot analyses (Figure S2) using paired primers and amplified probe sequences (Table S1). The hyphal growth and the colony morphology of the WT and each mutant strain were compared on potato dextrose agar (PDA), tryptone yeast-extract glucose agar (TYGA), and tryptone-glucose (TG) media. Deletion of *Aoras2* resulted in an abnormal growth pattern on PDA and TYGA media, and the average colony diameters of the WT and Δ*Aoras2* mutant strains on the PDA medium were 8.5 and 6.1 cm, respectively (Figures 1A and 1B). Meanwhile, *Aorheb* deletion caused abnormal mycelial growth on the TYGA medium and the average colony diameters of the WT and Δ*Aorheb* mutant strains were 8.5 and 5.9 cm, respectively (Figure 1B).

In addition, the deletion of Ras GTPase genes caused alterations in mycelial cell morphology. We observed that some mycelial cells of the Δ*Aoras2*, Δ*Aoras3*, and Δ*Aorheb* mutants (56%, 46%, and 26%, respectively) had swollen and irregular morphologies (Figure 1C). Upon staining the cell nucleus with 4ʹ,6-diamidino-2-phenylindole, multiple nuclei were clearly observed in the hyphal cells of the WT strain, whereas the nuclei of the mutants were difficult to stain as they appeared diffused and fragmented (Figure 1D). Degradation of chromosomal DNA is a prominent feature of cell apoptosis, which is a relatively common phenomenon (Nagata et al., 2003). Therefore, the fragmentation of chromosomal DNA in the mycelium of the WT and mutant strains was detected using the terminal deoxynucleotidyl transferase-mediated dUTP nick end-labeling (TUNEL) assay (Gavrieli et al., 1992), and the nuclei were stained using propidium iodide. Both the WT and mutant strains were stained with fluorescein isothiocyanate (FITC)-dUTP, and the nuclei of all mutants were observed to be in a diffuse form. Moreover, the FITC-dUTP fluorescence intensity (FI) of the mutants was higher than that of the WT strain (Figure 1D). As most of the DNA of the mutants was in a diffuse state, the FI ratios of FITC-dUTP and propidium iodide were calculated to analyze the differences in the extent of DNA damage and cell apoptosis between the WT and mutant strains. The FI ratios of WT and Δ*Aoras2*, Δ*Aoras3*, and Δ*Aorheb* mutant strains were 0.32, 0.54, 0.44, and 0.52, respectively (Figure 1E). Moreover, the *Aoras2*, *Aoras3*, and *Aorheb* gene transcripts showed a similar pattern, and their expression levels were upregulated during the conidiation stage (from day 3 to day 7), as compared with those in the vegetative growth stage (day 2). Among them, the expression of *Aoras2* was remarkably upregulated during the conidiation stage (Figure 1F).

### AoRas2, AoRas3, and AoRheb regulate conidiation and conidial germination

**Figure 2:**
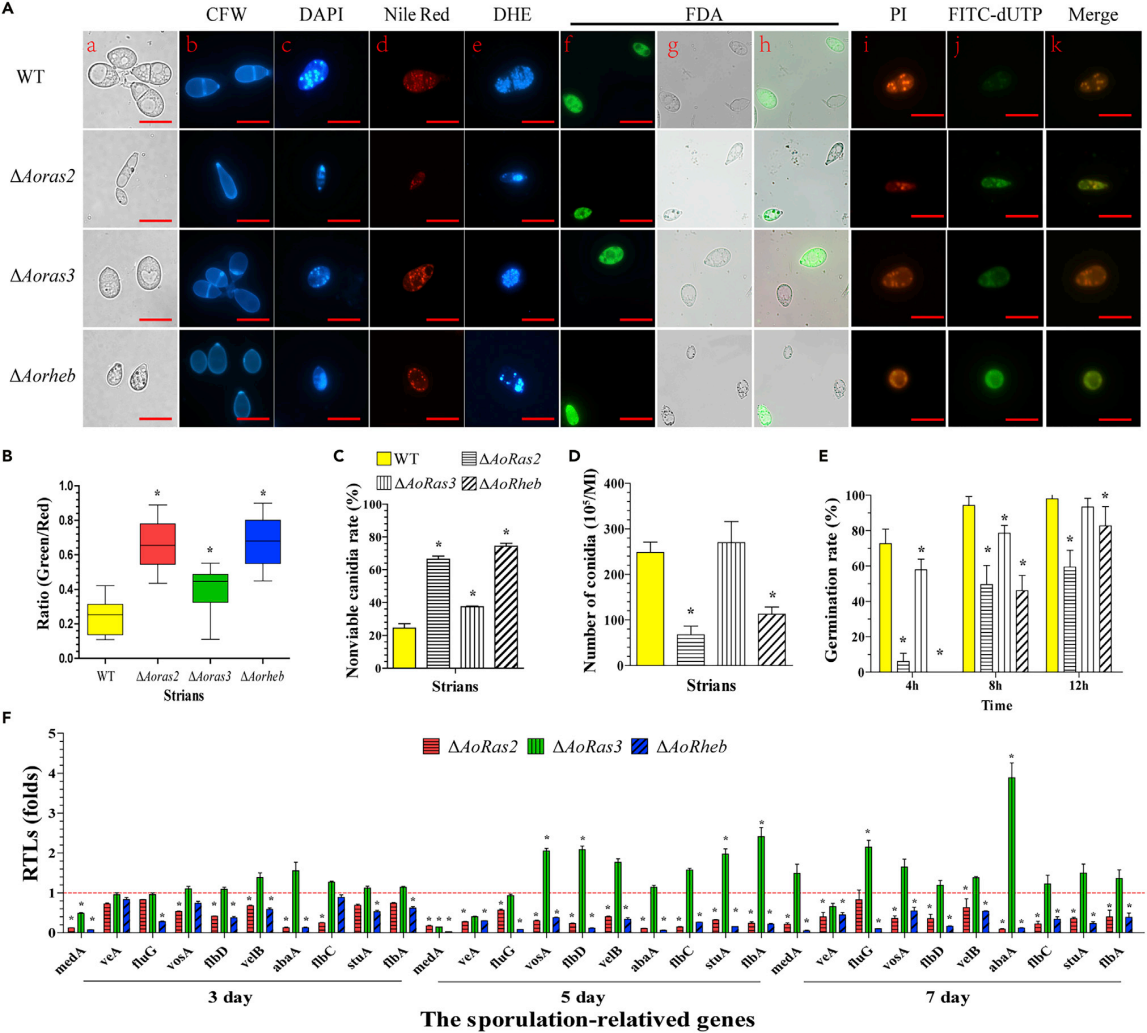
Comparison of conidiation, conidial germination, conidial activity, DNA fragmentation, and cell apoptosis between WT and mutant strains of *A. oligospora* (A) Conidia of WT and mutant strains were stained with CFW, DAPI, Nile Red, DHE, and FDA, followed by the TUNEL analysis. a. Conidia morphology of WT and mutant strains was observed under a light microscope. Conidia were stained with (b) CFW, (c) DAPI, (d) Nile Red, (e) DHE, and (f–h) FDA. The viable conidia stained with FDA emitted bright green fluorescence, whereas the nonviable ones did not show this effect. i–k. TUNEL analysis of spores. Samples were examined under a confocal laser scanning microscope. Scale bar, 10 μm. (B) Analysis of DNA fragmentation and cell apoptosis in the conidia. The ratio of green to red fluorescence intensity was determined for at least 30 fields viewed under a microscope, and the horizontal bars depict the median. The asterisk indicates a significant difference between the mutants and the WT strain (Tukey's HSD, p < 0.05). (C) Percentages of nonviable conidia of the WT and mutant strains. (D) Comparison of conidial yields between the WT and mutant strains. (E) Conidial germination rates in WT and mutant strains. (F) Relative transcription levels (RTLs) of sporulation-related genes in the mutant strain compared with those of the WT strain at different time points. The red line indicates the standard (which has an RTL of 1) for statistical analysis of the RTL of each gene in a deletion mutant compared with that in the WT strain under a given condition. Error bars in (C–F): Data are represented as mean ± SD. The asterisk in (C–F) indicates a significant difference between mutants and the WT strain (n = 3 for the WT strain (C–E), n = 9 for each mutant strain (C–E), n = 3 for each gene (F); Tukey's HSD, p < 0.05).

The spore yields of the WT and Δ*Aoras2*, Δ*Aoras3*, and Δ*Aorheb* mutant strains were 2.5, 0.7, 2.7, and 1.1 × 10^7^ spores/mL, respectively (Figure 2D). The conidia of the WT strain are usually obovoid in shape, with one septum formed near the base of the spore (Ma et al., 2020). In contrast, most conidia of the Δ*Aorheb* mutant were morphologically abnormal: 57.1% of the conidia lacked the septum and 67.1% of them were smaller than usual and round in shape and/or shrunk by 31.2% in size (Figure 2A, a, b). Similarly, 35.4% of the conidia of the Δ*Aoras3* mutant lacked the septum and 45.8% of the conidia were smaller and round in shape and/or shrunk by 27.6% in size. Most notably, the Δ*Aoras2* mutant exhibited severely deformed conidia that lacked septa (Figure 2A, a, b), and most of the conidia (71.7%) were narrow in shape and/or shrunk by 30.1% in size (Figure 2A, a, b), whereas other spores (28.3%) were small and narrow and shrunk by 62.8% in size. As in the hyphal cells, multiple nuclei were observed in the conidia of the WT and mutant strains (Figure 2A, c), and nuclear disintegration occurred in the spores of Δ*Aoras2*, Δ*Aoras3*, and Δ*Aorheb* mutants. The FI ratios of the WT and mutant strains (Δ*Aoras2*, Δ*Aoras3*, and Δ*Aorheb*) were 0.24, 0.66, 0.41, and 0.67, respectively, as estimated by the TUNEL assay (Figure 2A, c and 2B).

Furthermore, the deletion of Ras GTPase genes affected conidial germination. At 4 h, approximately 72.6% of spores germinated in the WT strain and 6.0% and 57.9% of spores germinated in the Δ*Aoras2* and Δ*Aoras3* mutants, respectively, whereas the spores of the Δ*Aorheb* mutant did not germinate at all (Figures 2E and S3E). Expression levels of 10 genes associated with sporulation were downregulated at each time point in the Δ*Aoras2* and Δ*Aorheb* mutants when compared with those in the WT strain. Specifically, expression levels of *abaA*, *flbA*, *flbC*, *flbD*, *fluG*, *medA*, *stuA*, *velB*, and *vosA* were significantly downregulated (p < 0.05) during the conidiation stage in the Δ*Aoras2* and Δ*Aorheb* mutants (Figure 2F). In contrast, expression levels of most genes were not significantly altered in the Δ*Aoras3* mutant, except for *medA* and *veA* genes, which were downregulated on days 5 and 7.

### AoRas2 and AoRheb regulate conidial activity and intracellular lipid storage

The conidial activity was analyzed by using fluorescein diacetate staining, and the unstained conidia were considered nonviable as they were either hollow with only shells or with granular residues present in their cells (Figure 2A, f–h). The percentages of nonviable conidia in the WT and mutant strains (Δ*Aoras2*, Δ*Aoras3*, and Δ*Aorheb*) were 24.5%, 66.4%, 37.4%, and 74.4%, respectively (Figure 2C). Moreover, we used Nile Red to visualize lipid droplets (LDs) in the fungal cells. Compared with their distribution in the WT cells, LDs were remarkably reduced and unevenly distributed in the Δ*Aoras2* and Δ*Aorheb* mutants, whereas there was no difference in the distribution between the Δ*Aoras3* mutant and the WT strain (Figure 2A, d).

### AoRas2, AoRas3, and AoRheb regulate stress response

**Figure 3:**
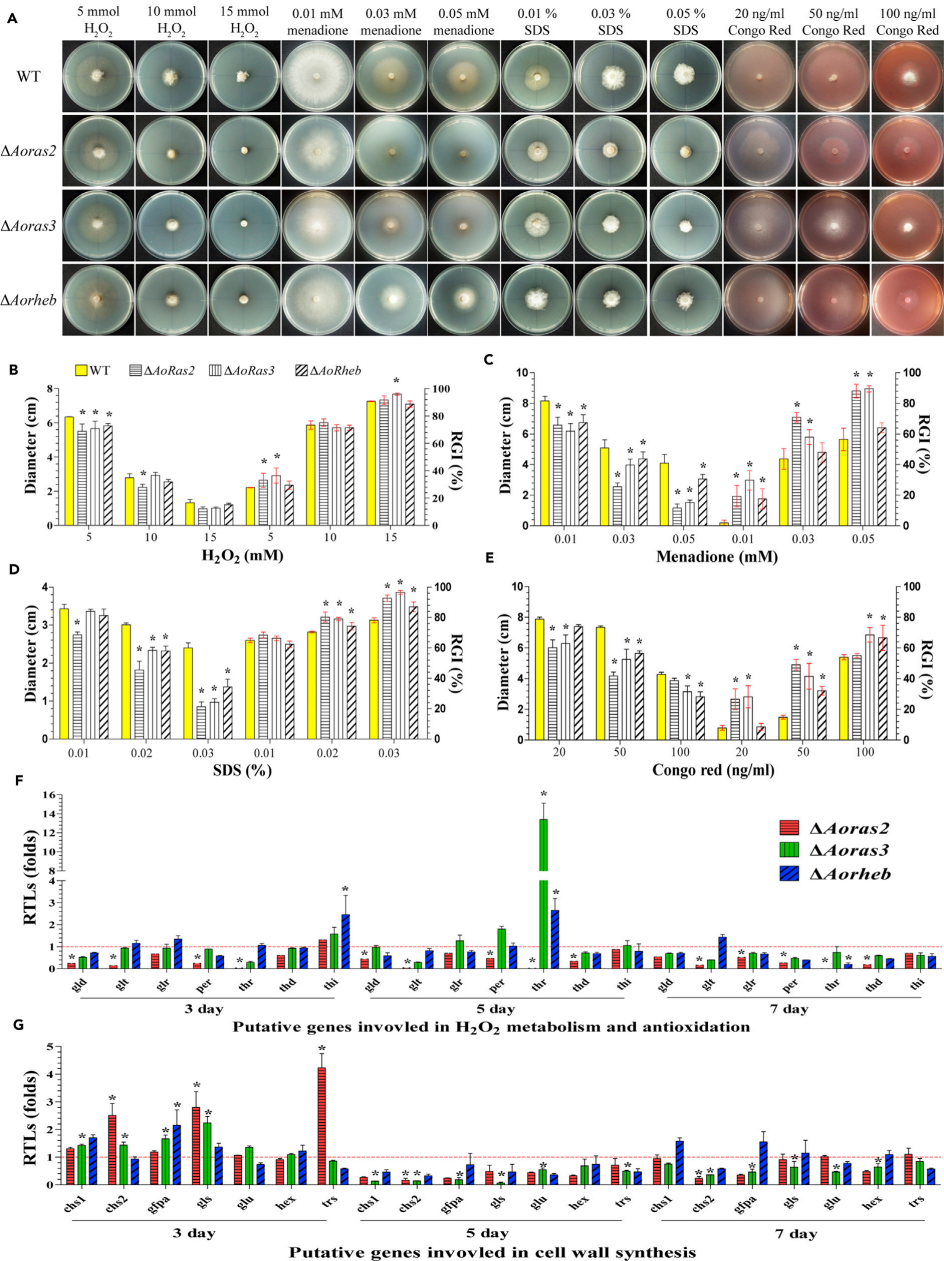
Comparison of the stress tolerance ability of strains to oxidative stress and cell wall-perturbing agents (A) Colony morphologies of the WT and mutant strains under oxidative stress conditions and in the presence of cell wall-perturbing agents. (B–E) Colony diameters and the relative growth inhibition (RGI) values of the strains cultured in the presence of (B) 5–15 mM H_2_O_2_, (C) 0.01–0.05 mM menadione, (D) 0.01–0.03% SDS, (E) 20–100 ng/mL Congo red. (F) Relative transcription levels (RTLs) of oxidation-related genes in the mutants when compared with the WT strain at different time points. (G) RTLs of cell wall synthesis-related genes in the mutants when compared with the WT strain at different time points. The red line indicates the standard (which has an RTL of 1) for the statistical analysis of the RTL of each gene in a deletion mutant compared with that in the WT strain under a given condition. Error bars in (B–G): Data are represented as mean ± SD. The asterisk in (B–G) indicates a significant difference between the mutants and the WT strain (n = 3 for the WT strain (B–E), n = 9 for each mutant strain (B–E), n = 3 for each gene (F, G); Tukey's HSD, p < 0.05).

The mutants were remarkably different from the WT strain in their sensitivities to chemical stressors. The mycelial growth of mutants was significantly inhibited on TG plates supplemented with H_2_O_2_ or menadione (Figure 3A). Particularly, the relative growth inhibition (RGI) values of the Δ*Aoras2* and Δ*Aoras3* mutants were significantly increased (p < 0.05) in the presence of H_2_O_2_ (5 mM) and menadione (0.01–0.05 mM) (Figures 3B and 3C). Similarly, the mutants showed differential sensitivity to cell wall-perturbing agents. The growth of all mutant strains was inhibited by sodium dodecyl sulfate (SDS) or Congo red, and the Δ*Aoras2* and Δ*Aoras3* mutants hardly grew on TG plates supplemented with 0.03% SDS (Figure 3A). The RGI values of the mutants were significantly increased (p < 0.05) in the presence of SDS (0.02%–0.03%) or Congo red (50 ng/mL) (Figures 3D and 3E). In addition, the growth of mutants was strongly inhibited on TG plates supplemented with 0.1 M NaCl or 0.25–0.50 M sorbitol, and their RGI values were significantly higher (p < 0.05) as compared with those in the WT strain (Figures S3A–S3C). Moreover, the Δ*Aoras2* and Δ*Aorheb* mutants were significantly inhibited (p < 0.05) at 38°C and were unable to grow properly at 42°C (Figures S3A and S3D).

To investigate the role of Ras GTPases in stress resistance, expression levels of seven genes, putatively involved in H_2_O_2_ metabolism and antioxidation, were analyzed. The expression of six genes (*gld*, *glt*, *glr*, *per*, *thr*, and *thd*) was downregulated in the Δ*Aoras2* mutant at the tested time points, and two genes, *glt* and *thr*, were hardly expressed at all. Similar to the observations in the Δ*Aoras2* mutant, expression levels of three genes, namely, *gld*, *glt*, and *thd*, were downregulated in the Δ*Aoras3* mutant at each time point. In contrast, the expression of *glr*, *per*, *thr*, and *thi* was upregulated in the Δ*AoRas3* mutant at some time points. Furthermore, the expression of two genes, *gld* and *thd*, was downregulated in the Δ*Aorheb* mutant at all tested time points, whereas expression levels of other genes were upregulated only at some time points. Of note, the expression of *thr* was considerably upregulated in the Δ*Aoras3* and Δ*Aorheb* mutants on day 5, implying that *thr* could be important in regulating the fungal response to oxidants mediated by AoRas3 and AoRheb (Figure 3F).

Similarly, expression levels of seven genes involved in cell wall synthesis were determined and compared between the WT and mutant strains. The expression of these genes was upregulated on day 3. In addition, expression levels of the genes *chs1*, *chs2*, *gfpa*, and *hex* were downregulated in Δ*Aoras2* and Δ*Aoras3* mutants on days 5 and 7, and the expression of the two genes, *chs2* and *glu*, was downregulated in the Δ*Aorheb* mutant at the tested time points (Figure 3G).

### Ras GTPases regulate the biocontrol potential of *A. oligospora*

**Figure 4:**
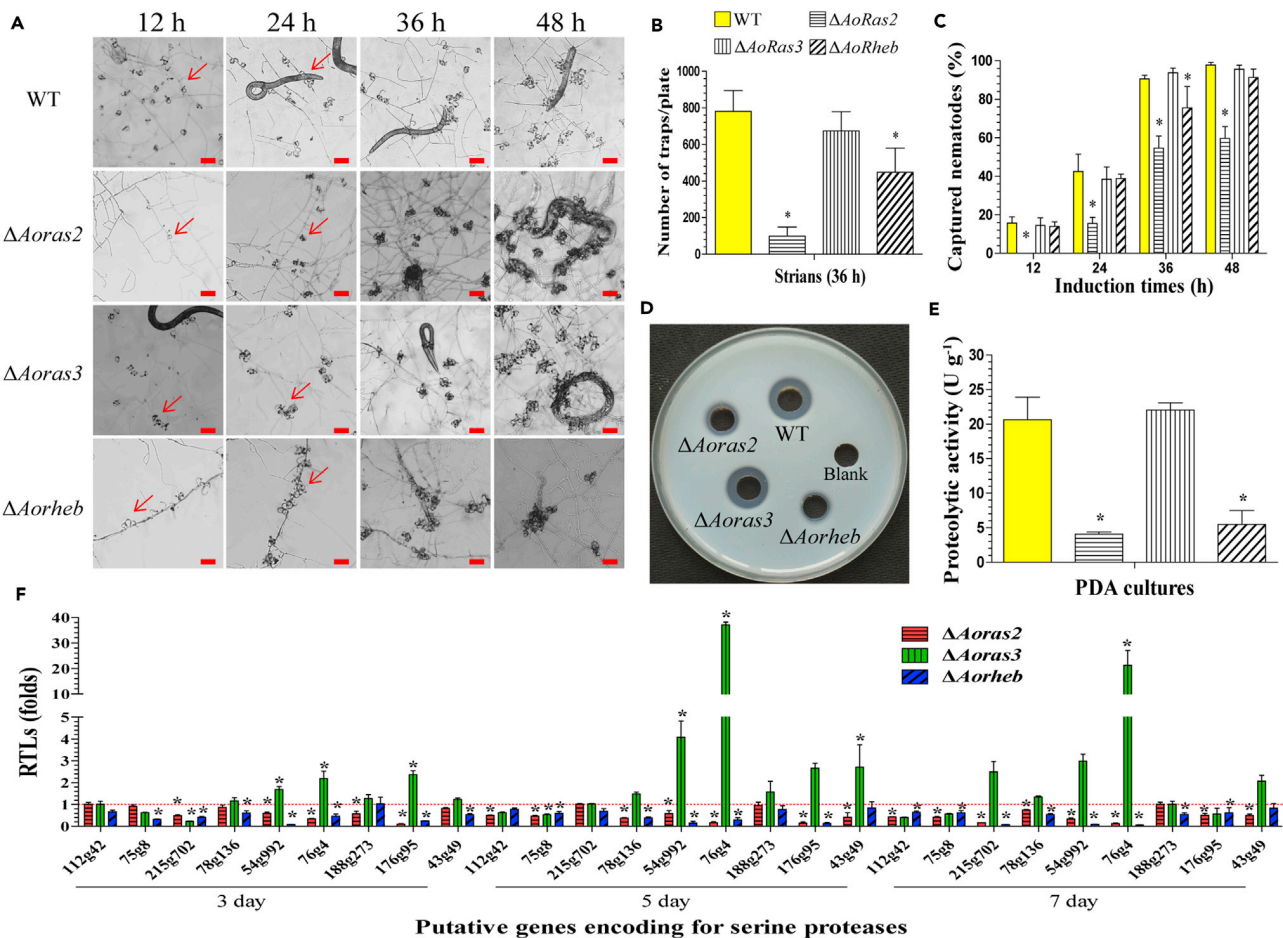
Comparison of trap formation, nematicidal activity, and extracellular proteolytic activity in the WT and mutant strains (A) Trap formation in the WT and mutant strains induced by nematodes at different time points. The red arrows point toward the traps produced by the WT strain and mutants. Scale bar, 100 μm. (B) The numbers of traps produced by the WT and mutant strains. (C) The percentage of captured nematodes at different time points. (D) Comparison of the extracellular proteolytic activities on casein plates. (E) Total extracellular protease activity of the WT and mutant strains exhibited on the 7-day-old PD broth. (F) Relative transcription levels (RTLs) of the genes encoding serine proteases in the mutants when compared with the WT strain at different time points. The red line indicates the standard (which has an RTL of 1) for the statistical analysis of the RTL of each gene in a deletion mutant compared with that in the WT strain under a given condition. Error bars in (B, C, E, F): Data are represented as mean ± SD. The asterisk in (B, C, E, F) indicates a significant difference between the mutants and the WT strain (n = 3 for the WT strain (B, C, E), n = 9 for each mutant strain (B, C, E), n = 3 for each gene (F); Tukey's HSD, p < 0.05).

Trap formation was observed on water agar plates in 12 h after the addition of nematodes (Figure 4A). The WT and mutant strains (Δ*Aoras3* and Δ*Aorheb*) produced immature traps containing one or two hyphal loops, and several immature traps containing one hyphal loop was produced by the Δ*Aoras2* mutant at 12 h. Subsequently, at 24 and 36 h, the WT and mutant strains formed mature traps containing five or more hyphal loops, whereas few mature traps were produced by the Δ*Aoras2* mutant (Figure 4B). At 48 h, almost all of the nematodes were captured by the WT (97.8%), Δ*Aoras3* (95.6%), and Δ*Aorheb* (91.4%) strains, whereas only 59.7% nematodes were captured by the Δ*Aoras2* mutant (Figure 4C).

Furthermore, we found that proteolytic activity of the Δ*Aoras2* and Δ*Aorheb* mutants was significantly decreased on casein plates (Figure 4D). In particular, proteolytic activity of the WT strain was 20.6 U/g hyphae, whereas those of Δ*Aoras2* and Δ*Aorheb* mutants were lower by 80.1% (4.1 U/g) and 73.4% (5.5 U/g) (Figure 4E), respectively. In addition, expression levels of six genes (*75g8*, *78g136*, *54g992*, *76g4*, *176g95*, and *43g49*), encoding serine proteases, were significantly downregulated in the Δ*Aoras2* mutant compared with that in the WT strain, whereas in the Δ*Aorheb* mutant, expression levels of seven genes (*112g42*, *75g8*, *215g702*, *78g136*, *76g4*, and *176g95*) were downregulated. Relative to the Δ*Aoras2* and Δ*Aorheb* mutants, the expression of only one gene (*75g8*) was downregulated in the Δ*Aoras3* mutant, and the expression of the *PII* gene (*76g4*) was considerably (37.1-fold) upregulated in the Δ*Aoras3* mutant compared with that in the WT strain (Figure 4F).

### Knockout of Ras GTPases affects intracellular cAMP levels and TOR and MAPK signaling

**Figure 5:**
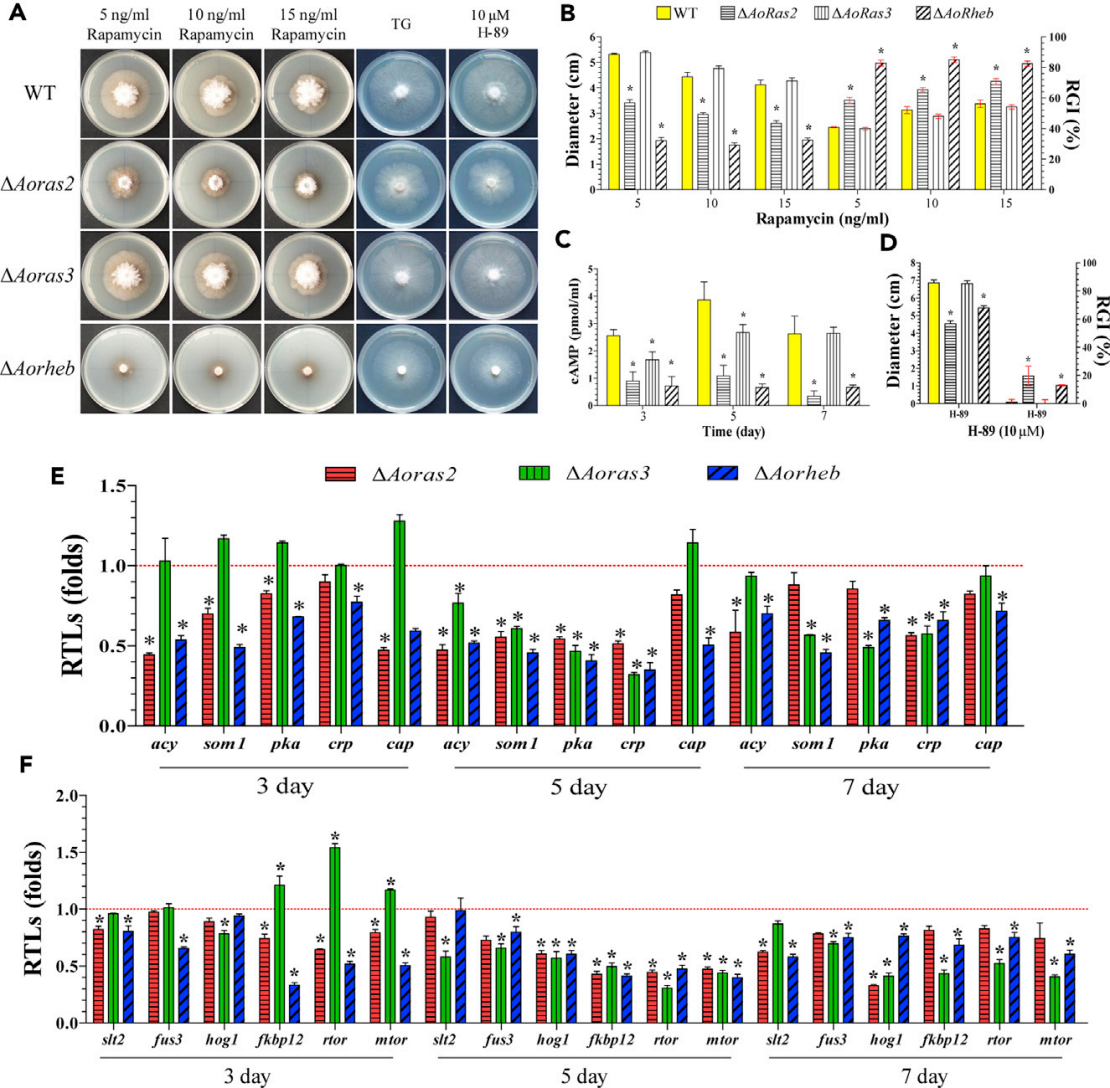
Comparison of rapamycin sensitivity and intracellular cAMP content in the WT and mutant strains (A) Colony morphologies of the WT and mutant strains cultured in the presence of 5–15 ng/mL rapamycin or 10 μM H-89. (B) Colony diameters and the relative growth inhibition (RGI) values of the WT and mutant strains cultured in the presence of 5–15 ng/mL rapamycin. (C) Comparison of intracellular cAMP content between the WT and mutant strains. (D) Colony diameters and RGI values of the WT and mutants cultured in the presence of 10 μM H-89. (E) Relative transcription levels (RTLs) of the cAMP/PKA signaling pathway-related genes in the mutants when compared with the WT strain at different time points. (F) RTLs of genes associated with the TOR and MAPK signaling pathways in the mutants when compared with the WT strain at different time points. The red line in (E and F) indicates the standard (which has an RTL of 1) for the statistical analysis of the RTL of each gene in a deletion mutant compared with that in the WT strain under a given condition. Error bars in (B–F): Data are represented as mean ± SD. The asterisk in (B–F) indicates a significant difference between the mutants and the WT strain (n = 3 for the WT strain (B–D), n = 9 for each mutant strain (B–D), n = 3 for each gene (E, F); Tukey's HSD, p < 0.05).

To determine the role of Ras GTPases in cAMP signaling of *A. oligospora*, the intracellular cAMP levels of the WT and mutants were analyzed. The cAMP levels in Δ*Aoras2* and Δ*Aorheb* mutants were significantly lower than in the WT strain (p < 0.05) at all tested time points and only on days 3 and 5 in the Δ*Aoras3* mutant (Figure 5D). The Δ*Aoras2* and Δ*Aorheb* mutants showed sensitivity to the PKA inhibitor H-89 at a concentration of 10 μM during the sensitivity tests (Figures 5A–5C). In addition, the Δ*Aorheb* mutant could hardly grow, and the growth of the Δ*Aoras2* mutant was inhibited on TG medium containing rapamycin (5–15 ng/mL), an inhibitor of the TOR signaling pathway. The RGI values of the WT (40.8%, 52.1%, and 56.2%) and the Δ*Aoras2* (58.6%, 65.4%, and 70.8%), Δ*Aoras3* (40%, 48%, and 54.2%), and Δ*Aorheb* (82.8%, 85.1%, and 82.5%) mutant strains were calculated for rapamycin at the concentrations of 5, 10, and 15 ng/mL, respectively (Figure 5B).

**Figure 6:**
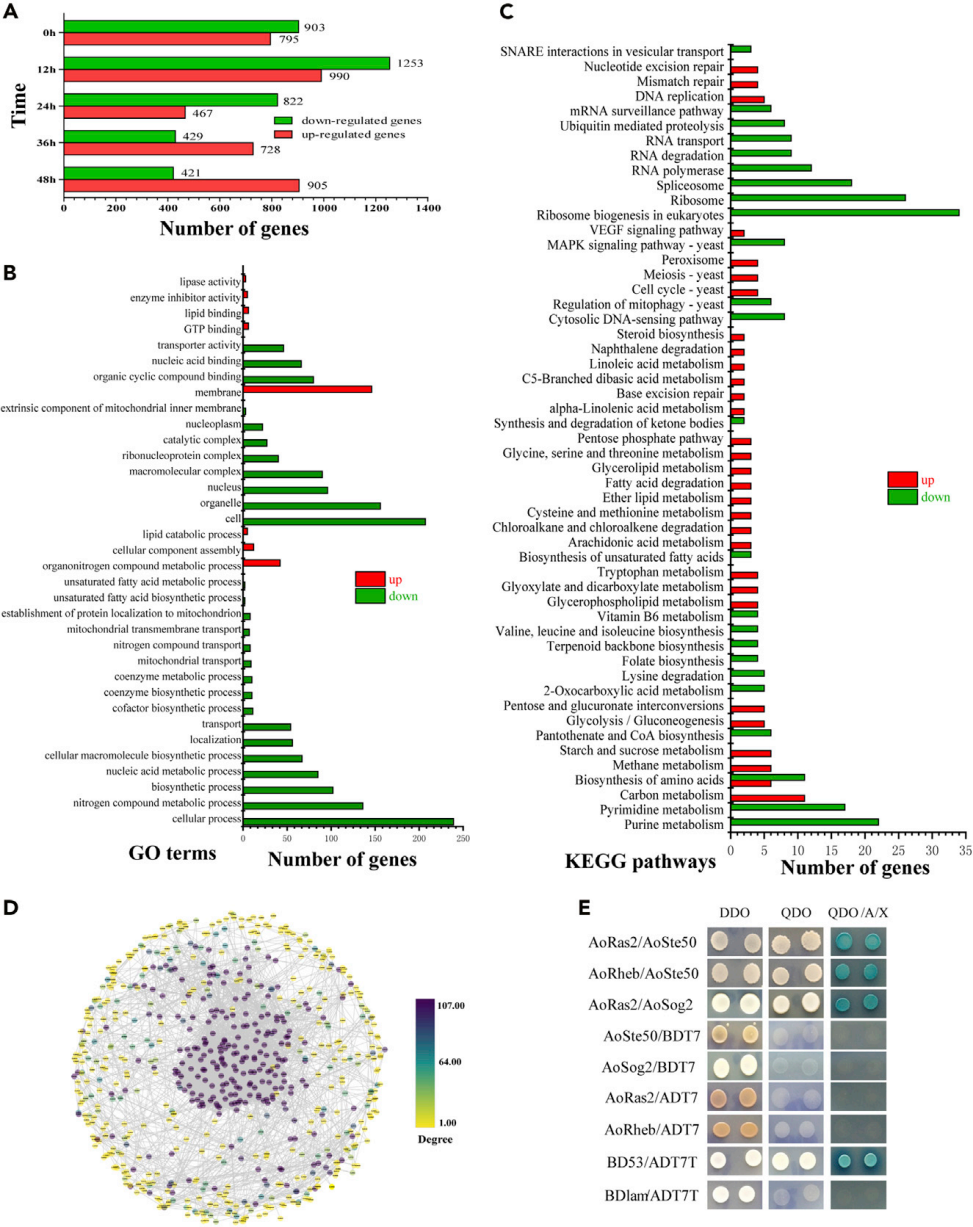
Analysis of the differentially expressed genes (DEGs) between the WT and Δ*Aoras2* mutant (A) The number of upregulated and downregulated DEGs at each time point. (B) Partial GO enrichment of DEGs at 12 h. (C) Partial KEGG enrichment of DEGs at 12 h. Red and green indicate upregulated and downregulated DEGs, respectively. (D) The association of DEGs in the Δ*Aoras2* mutant at every time point was analyzed through protein-protein interaction networks (STRING) and further visualized and analyzed with Cytoscape. (E) Yeast two-hybrid assay with AoRas2 or AoRheb as the bait and AoSte50 or AoSog2 as the prey. The interaction of pGBKT7-53 (BD53) and pGADT7-T (ADT7T) was used as the positive control, whereas that between pGBKT7-lam (BDlam) and ADT7T was used as the negative control.

In addition, the expression of the downstream genes of the cAMP/PKA, TOR, and MAPK pathways was significantly downregulated in the Δ*Aoras2* and Δ*Aorheb* mutants at the tested time points. In the Δ*Aoras3* mutant, some of the downstream genes were downregulated on days 5 and 7 (Figures 5E and 5F). Moreover, yeast two-hybrid (Y2H) assays confirmed the physical interaction of AoSte-50 with AoRas2 and AoRheB, respectively (Figure 6E).

### AoRas2 regulates global gene expression of *A. oligospora*

Mycelial samples of the WT and Δ*Aoras2* mutant strains were collected after trap formation induced by nematodes at different time points, and cDNA libraries of each sample were constructed and sequenced. High-throughput RNA sequencing data were obtained from three biological replicates maintained per strain. A range of clear reads (5.36–9.04 Gb) were obtained per sample. The percentage of Phred-like quality scores at the Q30 level (with an error probability of 1%) ranged between 98.6% and 98.8%, and the GC content was estimated to be between 46.5% and 48.0% (Table S2). The calculated Spearman correlation coefficient for the Δ*Aoras2* and WT strains ranged from 0.942 to 0.943, respectively (Figure S4A). The number of downregulated differentially expressed genes (DEGs) in the samples exceeded that of the upregulated DEGs at 0, 12, and 24 h, whereas the number of upregulated DEGs was higher at 36 and 48 h. The maximum number of DEGs, 2,240 (approximately 19.5% of the genome), was identified at 12 h of the experiment. Such a high number of DEGs revealed after *Aoras2* knockdown indicates that this gene is important for global gene activity of *A. oligospora* (Figure 6A).

Gene Ontology (GO) enrichment analysis was performed to analyze functional categories of these DEGs. At 0 h, 799 DEGs (372 upregulated and 427 downregulated) showed enrichment of 108 (57 upregulated versus 51 downregulated) GO terms (Table S3). A significant downregulation of a large number of genes related to carbohydrate metabolism, cellular amino acid metabolism, catalytic activity, and response to stimulus was observed. At 12 and 24 h, 1,273 (447 upregulated and 826 downregulated) and 685 (231 upregulated and 454 downregulated) DEGs showed enrichment of 160 (31 upregulated versus 129 downregulated) and 106 (60 upregulated versus 46 downregulated) GO terms, respectively (Figure 6B; Table S3). Furthermore, at 12 h, 136, 102, 56, and 54 DEGs involved in nitrogen compound metabolism, biosynthetic process, localization, and transport, respectively, were identified. At 24 h, the downregulation of DEGs involved in response to stimulus, signal transduction, and oxidoreductase activity was observed. Many genes, such as those encoding G protein-coupled receptors, serine/threonine kinases, succinate dehydrogenases, and isocitrate dehydrogenases, were suppressed during the initial stage of the trap formation in the mutant (12 and 24 h), so these genes may play an important role in trap formation in the WT strain (Tables S3 and S4). These genes are involved in signal transduction, energy production, and carbohydrate transport and metabolism, which play crucial roles in trap formation. At 36 and 48 h, 558 (346 upregulated and 212 downregulated) and 719 (450 upregulated and 269 downregulated) DEGs showed enrichment of 118 (52 upregulated and 66 downregulated) and 103 (49 upregulated and 54 downregulated) GO terms, respectively (Table S3). The DEGs revealed at these time points were mainly involved in fungal digestion and utilization of nematodes. Furthermore, expression levels of the pathogenicity-related genes, such as those associated with cytochrome P450 family and the synthesis of serine protease and polyketide synthase, were downregulated. Meanwhile, during the process of infection (12–48 h), the expression of genes encoding peroxisomal biogenesis-related proteins, which may be involved in the formation of dense bodies of trap cells in *A. oligospora*, was suppressed. In addition, expression levels of more genes involved in the biosynthesis of secondary metabolites, cytoskeleton assembling, cell division and cell cycle, cell wall synthesis, and mitochondrial biogenesis were suppressed. These analyses implied a critical role of AoRas2 in the transcriptional regulation of downstream genes that are essential for mycelial growth, development, and trap formation (Table S4).

In addition to the analyses using GO classification, we found that at 0 h, 186 DEGs (123 downregulated and 63 upregulated) were significantly enriched in 50 (35 downregulated and 20 upregulated) Kyoto Encyclopedia of Genes and Genomes (KEGG) pathways (Table S5). In addition, many downregulated genes were enriched in carbohydrate and amino acid metabolism pathways. Moreover, genes involved in cell cycle, MAPK signaling pathway, and longevity-regulating pathway (LRP) were all downregulated. At 12 and 24 h, 366 (275 downregulated and 91 upregulated) and 195 (133 downregulated and 62 upregulated) DEGs were significantly enriched in 53 (24 downregulated and 30 upregulated) and 37 (26 downregulated and 14 upregulated) KEGG pathways, respectively (Figure 6C; Table S5). Similarly, expression levels of several genes related to the MAPK signaling pathway were downregulated at the aforementioned two points. At 36 and 48 h, 116 (72 downregulated and 44 upregulated) and 186 DEGs (100 downregulated and 86 upregulated) were significantly enriched in 29 (18 downregulated and 13 upregulated) and 53 (29 downregulated and 28 upregulated) KEGG pathways, respectively (Table S5). At these two points, expression levels of several LRP-related genes were downregulated. Meanwhile, expression levels of several genes associated with mitophagy were downregulated at 12, 24, and 36 h, and the expression of the AMP-activated kinase (AMPK) signaling-related genes was downregulated at 12, 24, 36, and 48 h.

The association of DEGs in the Δ*Aoras2* mutants at each time point was analyzed through the protein-protein interaction network downloaded from the STRING database. In this network, AoRas2 interacted with the Ras GTPase-activating protein AoCla2 (173g249), never in mitosis gene A-related kinase (AOL_s00176g88), cell morphogenesis protein AoSog2 (AOL_s00188g20), and AoKel2 (AOL_s00193g134). The physical interaction of AoRas2 with AoSog2 was confirmed through the Y2H assay (Figures 6D and 6E; Table S6).

### AoRas2 and AoRheb play important roles in maintaining morphology and activity of mitochondria

**Figure 7:**
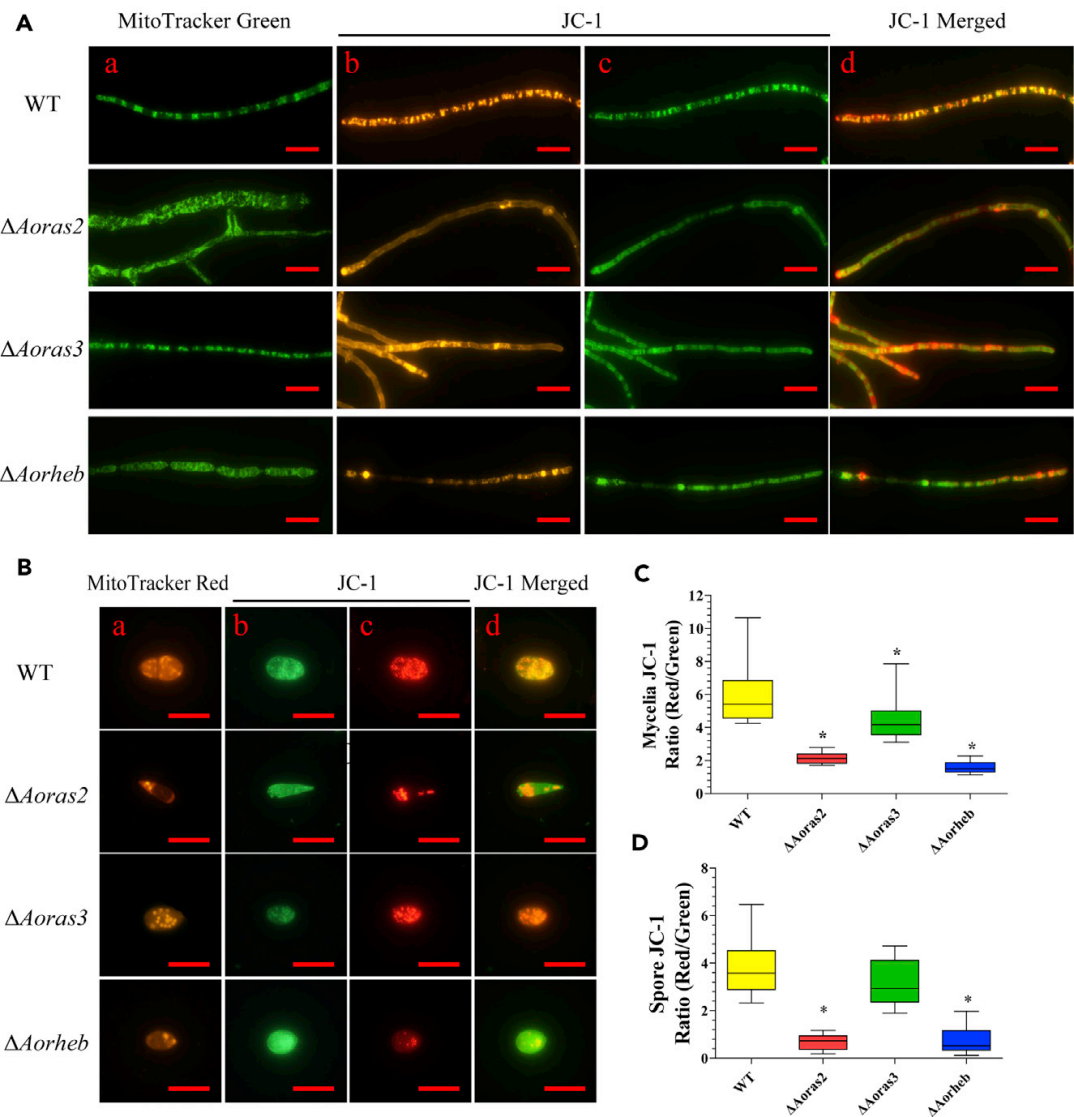
Effect of AoRas2 and AoRheb on mitochondrial morphology and activity (A) Mitochondrial morphology and mitochondrial membrane potential (MMP) of mycelia were compared between the WT and mutant strains. a. Mycelia were stained with MitoTracker Green. b–d. MMP was analyzed through tetraethylbenzimidazolyl-carbocyanine iodide (JC-1) staining. With JC-1 staining, normal mitochondria mainly yield red fluorescence due to high MMP, whereas abnormal mitochondria mainly yield green fluorescence due to low MMP. (B) Mitochondrial morphology and MMP of conidia were compared between the WT and mutant strains. a. Spores were stained with MitoTracker Red CMXRos, which can specifically label bioactive mitochondria. b–d. MMP was analyzed through JC-1 staining. Samples were examined under a confocal laser scanning microscope. Scale bar, 10 μm. (C and D) MMP level analysis in (C) mycelia and (D) conidia. The ratio of red to green fluorescence intensity was obtained for at least 30 fields viewed under a microscope, and the horizontal bars depict the median. The asterisk in (C and D) indicates a significant difference between the mutants and the WT strain (Tukey's HSD, p < 0.05).

A large number of mitochondrion-related genes were differentially expressed in the transcriptome. Compared with the WT, the mycelia of the Δ*Aoras2* and Δ*Aorheb* mutants displayed several giant mitochondria owing to the imbalance between mitochondrial fusion and fission (Figure 7A, a). In addition, the number of bioactive (high-functioning) mitochondria in the spores of Δ*Aoras2* and Δ*Aorheb* mutants was reduced and unevenly distributed (Figure 7B, a). Moreover, we determined mitochondrial activity using tetraethylbenzimidazolyl-carbocyanine iodide (JC-1), a cationic dye that accumulates in energized mitochondria (Reers et al., 1995). The mycelia and spores of Δ*Aoras2* and Δ*Aorheb* mutants displayed stronger green FI than those of the WT fungi. The ratio of the red/green FI was significantly decreased in the Δ*Aoras2* and Δ*Aorheb* mutants when compared with the WT strain (Figure 7A-b–d, B-b–d). In the hyphae, the FI ratios of WT and mutant strains (Δ*Aoras2*, Δ*Aoras3*, and Δ*Aorheb*) were 5.95, 2.14, 4.63, and 1.56, respectively (Figure 7C), whereas in the spores, they were estimated to be 3.78, 0.65, 3.16, and 0.71, respectively (Figure 7D).

### AoRas2 and AoRheb regulate reactive oxygen species levels and autophagy in the mycelium and spores

We compared reactive oxygen species (ROS) levels in WT and mutant strains by using dihydroethidium staining. ROS were aggregated in certain regions of the mycelium in the WT strain, whereas ROS were scattered and dispersed in the mycelia of mutants. The FI values for ROS in the WT and mutant strains (Δ*Aoras2*, Δ*Aoras3*, and Δ*Aorheb*) were 38.8, 52.7, 44.6, and 59.6, respectively (Figures S5A and S5D). The conidia of the WT and Δ*Aoras3* mutant strains produced high levels of ROS, whereas the conidia of the Δ*Aoras2* and Δ*Aorheb* mutants produced low levels of ROS, as they contained few bioactive mitochondria upon which the distribution of ROS depends (Figure S5B and S5C). The FI values of the conidia in WT and mutant strains (Δ*Aoras2*, Δ*Aoras3*, and Δ*Aorheb*) were 106.9, 55.6, 104.2, and 50.3, respectively (Figure S5E).

**Figure 8:**
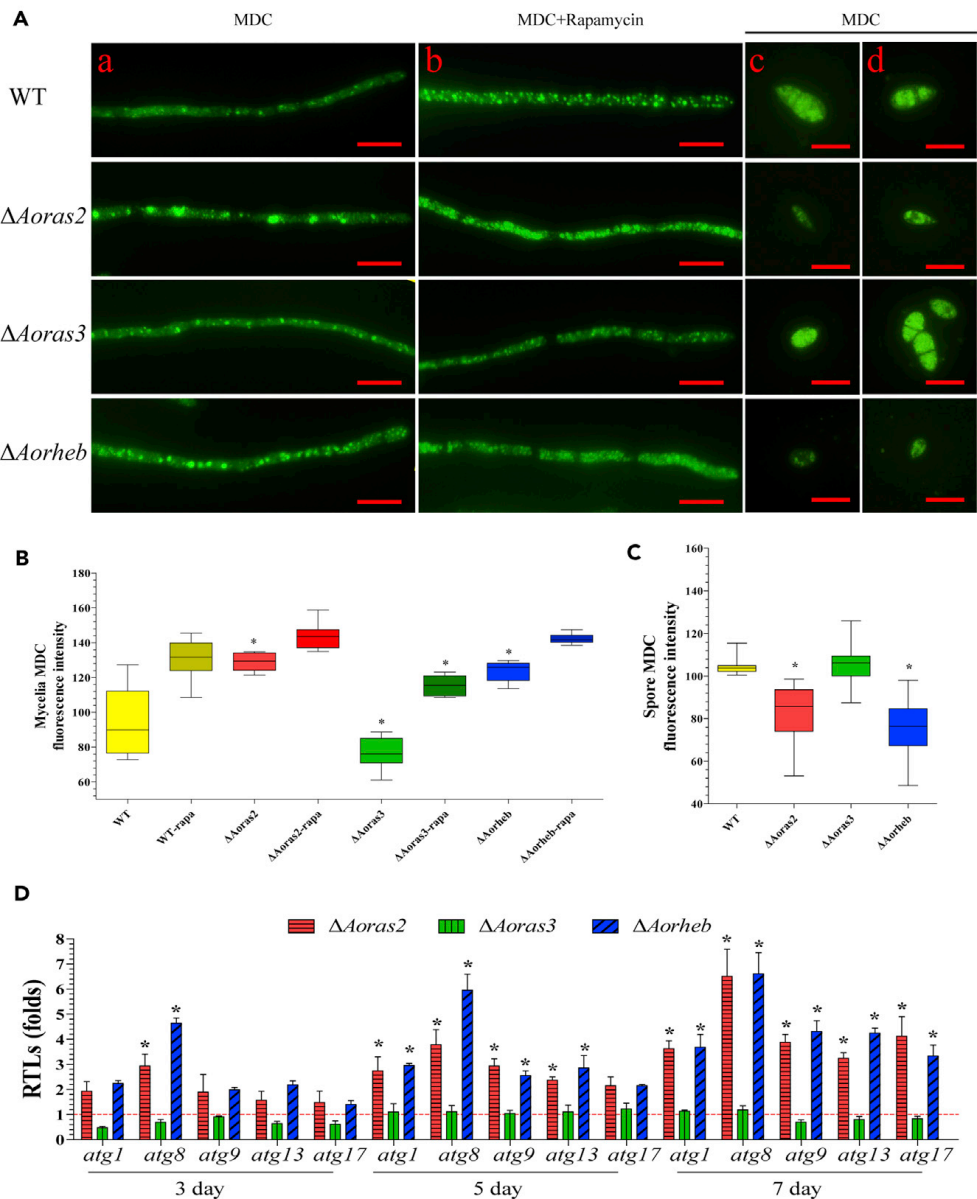
Effect of AoRas2 and AoRheb on autophagy in mycelia and spores (A) Mycelia and spores of the WT and mutants were stained with monodansylcadaverine (MDC). a–b. The autophagosomes in mycelium. MDC + rapamycin, the sample was treated with rapamycin for 10 min. c–d. The autophagosomes in spores. The samples were examined under a confocal laser scanning microscope. Scale bar, 10 μm. (B, C) The autophagic activity of the WT and mutant strains was analyzed by estimating the MDC fluorescence intensity values of (B) hyphae and (C) spores. The autophagic activity was analyzed by obtaining the fluorescence intensity values for at least 30 fields viewed under a microscope, and the horizontal bars depict the median. The asterisk in (B, C) indicates a significant difference between the mutants and the WT strain (Tukey's HSD, p < 0.05). (D) Relative transcription levels (RTLs) of autophagy-related genes were determined at different time points. The red line indicates the standard (which has an RTL of 1) for the statistical analysis of the RTL of each gene in a deletion mutant compared with that in the WT strain under a given condition. Error bars: Data are represented as mean ± SD. The asterisk indicates a significant difference between the mutants and the WT strain (n = 3 for each gene; Tukey's HSD, p < 0.05).

To evaluate autophagy, the mycelium and spores of WT and mutant strains were stained with monodansylcadaverine. The Δ*Aoras2* and Δ*Aorheb* mutants produced more autophagosomes in the hyphae than did WT and Δ*Aoras3* mutant strains. The FI values of the WT and mutant strains (Δ*Aoras2*, Δ*Aoras3*, and Δ*Aorheb*) were 94.1, 128.6, 76.3, and 123.2, respectively (Figures 8A and 8B). In contrast, the conidia of the Δ*Aoras2* and Δ*Aorheb* mutants had fewer autophagosomes than WT and Δ*Aoras3* mutant strains. The FI values of the conidia in WT and mutant strains (Δ*Aoras2*, Δ*Aoras3*, and Δ*Aorheb*) were estimated to be 104.6, 82.4, 105.6, and 75.6, respectively (Figure 8C). Expression levels of five genes (*atg1*, *atg8*, *atg9*, *atg13*, and *atg17*) encoding autophagy-related proteins were upregulated in the mycelium of the Δ*Aoras2* and Δ*Aorheb* mutants, whereas in the Δ*Aoras3* mutant, their expression was downregulated on day 3, and not significantly different (p > 0.05) from that in the WT strain on days 5 and 7 (Figure 8D).

### AoRas2 and AoRheb regulate secondary metabolite production

**Figure 9:**
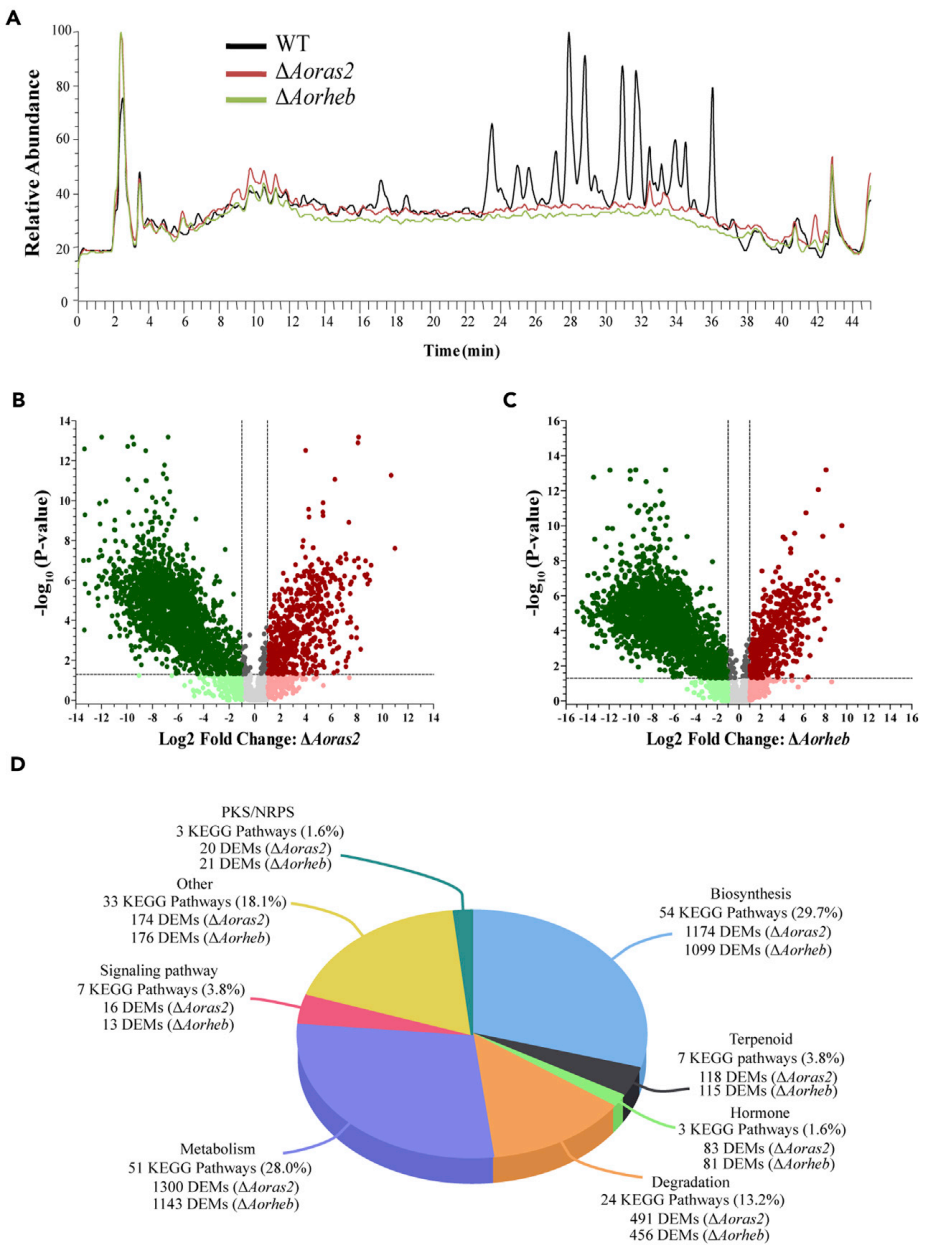
Effect of AoRas2 and AoRheb on secondary metabolites produced by *A. oligospora* (A) Comparison of HPLC profiles of the WT and mutant strains (Δ*Aoras2* and Δ*Aorheb*). (B) The number of differentially expressed metabolites (DEMs) in the Δ*Aoras2* mutant. (C) The number of DEMs in the Δ*Aorheb* mutant. Green dots suggest downregulated DEMs, whereas red dots suggest upregulated DEMs. (D) The number of KEGG pathways and DEMs in the Δ*Aoras2* and Δ*Aorheb* mutants.

We further compared metabolic profiles of WT and mutant strains (Δ*Aoras2* and Δ*Aorheb*) by using liquid chromatography-mass spectrometry to elucidate the role of AoRas2 and AoRheb in the secondary metabolism of *A. oligospora*. High-performance liquid chromatography analysis revealed that metabolic profiles of the Δ*Aoras2* and Δ*Aorheb* mutants were significantly different from that of the WT strain, and the abundance of many metabolites was decreased in the two mutants (Figure 9A).

Untargeted metabolomics was performed to further analyze metabolites of the Δ*Aoras2* and Δ*Aorheb* mutants. Overall, 3,431 metabolites were annotated in all samples (Table S7) and clearly separated into three groups according to the principal component analysis (Figure S6A). The numbers of upregulated and downregulated metabolites identified in the Δ*Aoras2* and Δ*Aorheb* mutants compared with those in the WT strain were 569 and 2,076, and 443 and 2,230, respectively (Figure 9B and 9C). These differentially expressed metabolites (DEMs) were categorized into 182 KEGG pathways (Table S8), which were further classified into biosynthesis (54), terpenoids (7), hormones (3), degradation (24), metabolism (51), signaling pathways (7), polyketide synthase-nonribosomal peptide synthetases (3), and others (33) (Figure 9D, S6C, and S6D; Table S8). Among them, we found that the content of arthrobotrisin (C_22_H_34_O_6_), a specific metabolite of *A. oligospora* (Wei et al., 2011), was downregulated, respectively, by 9.57- and 9.95-fold in the Δ*Aoras2* and Δ*Aorheb* mutants as compared with that in the WT strain. Moreover, some DEMs related to TOR signaling, AMPK signaling, and LRP were produced in the Δ*Aoras2* and Δ*Aorheb* mutants. For example, the amounts of hexose, DL-arginine, L-isoleucine, and L-norleucine were higher, whereas the amounts of L-(+)-leucine, trioxsalen, and *trans*-resveratrol were lower in the Δ*Aoras2* and Δ*Aorheb* mutants compared with those in the WT strain. In addition, the Δ*Aoras2* and Δ*Aorheb* mutants showed an increased amount of gamma-aminobutyric acid (GABA), which affects the ability of the GABA receptor to suppress adenylate cyclase expression, thus affecting the cAMP/PKA signaling pathways (Terunuma, 2018) (Figure S6C and S6D; Table S8).

## Discussion

Ras GTPases play a central role in sensing and responding to environmental cues in all eukaryotes (Goitre et al., 2014). In this study, three Ras GTPases were identified in the NT fungus *A. oligospora*, and we found that Ras GTPases, particularly AoRas2 and AoRheB, play vital roles in vegetative growth, conidiation, trap formation, DNA damage, mitochondrial activity, ROS levels, lipid storage, autophagy, and secondary metabolism of *A. oligospora*. Of importance, our transcriptomic analysis (12 h) demonstrated that nearly one-fifth of all *A. oligospora* genes are transcriptionally regulated by AoRas2 in a direct or indirect manner, which enables understanding of the pleiotropic effects of Ras GTPases, as discussed below.

Ras GTPases regulate hyphal growth, morphology, and stress resistance in fungi. For example, disruption of *ras2* in *F. graminearum* led to morphological defects, such as slow growth and abnormal colony formation (Bluhm et al., 2007). Ras2 regulates the apical growth of hyphae and cell wall synthesis in *N. crassa* (Kanauchi et al., 1997), whereas the disruption of *ras3* in *Beauveria bassiana* led to increased sensitivity to NaCl, menadione, H_2_O_2_, Congo red, and high temperature (Guan et al., 2015). In this study, deletion of *Aoras2* resulted in the reduction of mycelial growth, and some mycelial cells of the Δ*Aoras2*, Δ*Aoras3*, and Δ*Aorheb* mutants became swollen and exhibited irregular morphologies. Moreover, deletions of *Aoras2*, *Aoras3*, and *Aorheb* conferred sensitivity to several chemicals, which correlated with transcriptional repression of the expression of 13 genes associated with antioxidant processes and cell wall synthesis. Furthermore, the Δ*Aoras2* and Δ*Aorheb* mutants showed significant sensitivity to heat shock, and the expression of genes encoding heat shock proteins were significantly downregulated in the transcriptome of the Δ*Aoras2* mutant strain. These results showed that the function of Ras GTPases is conserved in fungal growth, cell development, and stress tolerance.

Apart from growth, Ras GTPases also play a role in spore production and development. For example, disruption of *ras2* or *ras3* in *F. graminearum*, *B. bassiana*, and *N. crassa* delayed spore germination and conidial formation (Bluhm et al., 2007; Kanauchi et al., 1997; Guan et al., 2015). In this study, deletion of *Aoras2* and *Aorheb* caused a reduction in the conidial yield and delayed spore germination and led to abnormal spore morphology. Correspondingly, the expression of sporulation-related genes, such as *abaA*, *flbA*, *fluG*, *stuA*, and *velB*, was substantially downregulated in the Δ*Aoras2* and Δ*Aorheb* mutants during the conidiation stage. Our recent studies showed that *stuA* and *velB* are essential for conidiation of *A. oligospora* (Xie et al., 2019; Zhang et al., 2019a, 2019b) and ROS are required for conidial germination in this fungus (Li et al., 2017). Moreover, LDs act as important cellular energy resources and participate in fungal spore development (Fan et al., 2015; Lin et al., 2013; Ren et al., 2014). In the Δ*Aoras2* and Δ*Aorheb* mutant strains, the number of active spores as well as intracellular LDs and ROS levels were lower than in the WT strain. In addition, we also found that the deletion of *Aoras2*, *Aoras3*, or *Aorheb* led to DNA damage and apoptosis in the mycelium and spores. Our results showed reduced or delayed spore germination of the Δ*Aoras2* and Δ*Aorheb* mutants, which may be associated with dysregulated spore activity, decreased ROS levels, lower intracellular lipid storage, and apoptosis.

*A. oligospora* is a typical species of NT fungi; therefore, traps and extracellular serine proteases are important for its activity of nematode biocontrol. The Δ*Aoras2* and Δ*Aorheb* mutants produced fewer traps, and their extracellular proteolytic activities were significantly lower than in the WT strain. In addition, expression levels of several serine protease genes were significantly downregulated in the mutants. These results are consistent with those of previous studies conducted on other pathogenic fungi. For example, lower virulence was observed in the Δ*ras2* and Δ*ras3* mutants of *B. bassiana* (Guan et al., 2015; Xie et al., 2013) and in the Δ*rhbA* mutant of *Aspergillus fumigatus* (Panepinto et al., 2003). Thus, Ras GTPases play a crucial role in the pathogenicity of various fungi. Furthermore, AoRas2 and AoRheb regulate trap formation and production of pathogenicity-related proteins, such as serine proteases, for nematode predation.

Ras GTPases activate the MAPK and TOR signal transduction pathways in fungi (Lee and Kronstad, 2002; Masuda et al., 1995; Tsao et al., 2009; Weeks and Spiegelman, 2003). In *S. cerevisiae*, for instance, Ras2 regulates the production of cAMP by adenylyl cyclase and the activation of the MAPK pathway during filamentous growth (Mosch et al., 1999). In this study, we used multiple methods to analyze the relationship between Ras GTPases and the TOR, MAPK, and PKA signaling pathways. For example, intracellular cAMP levels, which determine the activity of the cAMP/PKA pathway, were significantly reduced in the Δ*Aoras2* and Δ*Aorheb* mutants. Meanwhile, the Δ*Aoras2* and Δ*Aorheb* mutants showed a sensitivity to H-89 and rapamycin, which indicated that the activity of the PKA and TOR signaling pathways, respectively, was decreased or lost in the mutant strains. Therefore, AoRas2 and AoRheb are most likely involved in the regulation of the PKA and TOR signaling pathways. Moreover, the physical interactions of AoRas2 and AoRheb with AoSte50, and of AoRas2 with AoSog2 were verified by the Y2H assays. In *Schizosaccharomyces pombe*, Sog2 forms a complex with Nak1 (Ste20-like kinases), which regulates cell morphology, polarity, and growth (Cipak et al., 2013; Huang et al., 2005). Ste50 and Ste20 function upstream of the MAPK signaling to activate it (Saito, 2010). Furthermore, the expression of the downstream genes of the MAPK, PKA, and TOR pathways was significantly downregulated in the Δ*Aoras2* and Δ*Aorheb* mutants. Similarly, genes encoding proteins downstream of MAPK were also downregulated in the Δ*Aoras3* mutant. In addition, many downregulated DEGs identified through the transcriptional analysis in the Δ*Aoras2* mutant were involved in the TOR, MAPK, and AMPK signaling pathways. Similarly, some DEMs in the metabolome of the Δ*Aoras2* and Δ*Aorheb* mutants were involved in the aforementioned pathways and in the cAMP signaling pathway. These results indicate that AoRas2 and AoRheb are involved in the TOR and MAPK signaling pathways and affect cAMP/PKA signaling by maintaining cellular cAMP levels in *A. oligospora*.

Transcriptional analysis of the WT strain and *Aoras2* deletion mutant showed that more than 19% of all genes are regulated by AoRas2 at the early stage of trap formation (12 h). This genome-wide regulatory role of AoRas2 helps to understand the pleiotropic effects of Ras GTPases in *A. oligospora*. GO functional annotation revealed that many genes downregulated in the Δ*Aoras2* mutant are involved in the metabolism of carbohydrates, amino acids, and lipids. These genes were also involved in biosynthesis, transport, and catabolism of secondary metabolites and in mitochondrial functioning. Mitochondria represent a dynamic functional system that maintains homeostasis through continuous fusion and fission events as well as via the autophagic turnover (Haeussler et al., 2020). Mitochondria are essential organelles for many fundamental cellular processes, including anabolic and catabolic phenomena, energy production, lipid metabolism, redox signaling, and programmed cell death (Kou et al., 2019). In this study, the mitochondrial activity, ROS levels, autophagy, apoptosis, and metabolic profiling of the Δ*Aoras2* and Δ*Aorheb* mutants showed significant differences compared with the analogous parameters of the WT strain. In summary, the deletion of *Aoras2* and *Aorheb* contributed to the alterations in ROS levels and autophagy in the mycelium and spores due to mitochondrial dysfunction, which may be caused by unequal division of mitochondria during the genetic process. Such mitochondrial dysregulation led to decreased metabolite synthesis, cell apoptosis, and DNA damage. Moreover, several genes encoding proteins involved in the AMPK (n = 7 genes), LRP (n = 10), MAPK (n = 11), and TOR (n = 3) signaling pathways, as well as in mitophagy (n = 6) and cell cycle (n = 7), were downregulated in the transcriptome at 12 h. Expression levels of these genes were further confirmed by reverse transcription quantitative PCR (RT-qPCR) (Table S9), which suggested that AoRas2 plays an important role in modulating the phenotypic traits of *A. oligospora*.

**Figure 10:**
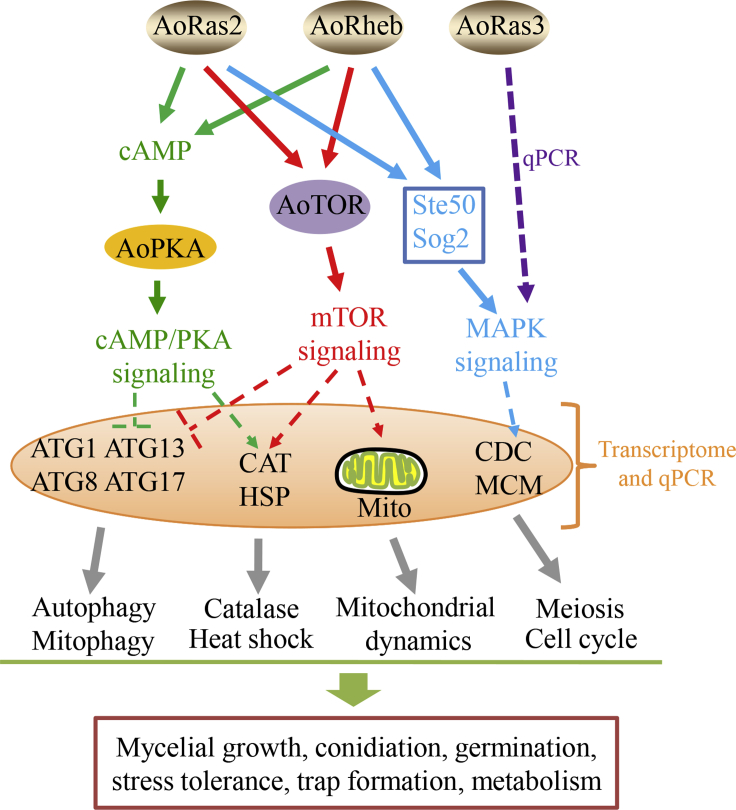
Proposed model of the regulation of cellular processes in *A. oligospora* by Ras GTPases Ras GTPases function upstream of various signaling pathways, such as cAMP/PKA, TOR, and MAPK, thereby further affecting multiple cellular and biological processes. cAMP, cyclic adenylic acid; AoPKA, protein kinase A in *A. oligospora*; AoTOR, TOR in *A. oligospora*; qPCR, reverse transcription qualitative PCR; Mito, mitochondria; Ste50, protein Ste50; Sog2, protein Sog2; ATG1, ATG8, ATG13, and ATG17, autophagy-related proteins; CAT, catalase; HSP, heat shock protein; CDC, cell division cycle; MCM, minichromosome maintenance protein.

Based on the combined results of the phenotypic traits, RT-qPCR data, and transcriptional analysis, we propose the following model for Ras GTPases that affect the biological functions in *A. oligospora* (Figure 10). Ras GTPases (AoRas2, AoRas3, and AoRheb) function upstream of the signaling pathways, such as cAMP/PKA, TOR, and MAPK. Activated PKA and TOR pathways inhibit the expression of autophagy-related genes (*atg1*, *atg8*, *atg13*, and *atg17*) and induce the expression of catalase and heat shock proteins, thereby affecting autophagy, antioxidant processes, and heat shock. Moreover, the MAPK signaling pathways activate the expression of downstream cell division cycle and minichromosome maintenance protein to regulate cell division and cell cycle. In addition, TOR signaling disturbs mitochondrial dynamics. Overall, our results provide systematic insights into the common and unique roles of Ras GTPases in *A. oligospora*, which not only regulate hyphal growth, conidiation, and multi-stress tolerance but also contribute to trap formation and virulence. Of importance, Ras GTPases also affect autophagy, mitochondrial activity, and secondary metabolism in this fungus. In summary, this study expands our understanding of the biological functions of Ras GTPases in *A. oligospora*, thereby providing a foundation for unveiling the mechanism of lifestyle switching in NT fungi and for exploring potential application of *A. oligospora* in the biocontrol of pathogenic nematodes.

### Limitations of the study

NT fungi form unique infection structures to capture and kill free-living nematodes. In this study, we characterized three orthologous Ras GTPases in a typical NT fungus species. It is difficult to construct a double- or multi-knockout mutant in *A. oligospora* (ATCC24927) owing to the lack of effective selection markers. Therefore, in the future, it is necessary to develop a highly effective method for gene editing to allow further investigation of the interaction between different Ras GTPases. Moreover, the proposed model for the regulation of the biological functions of *A. oligospora* by Ras GTPases is mainly generated from the expression level changes of genes involved in signaling pathways (e.g., PKA, TOR, and MAPK signaling) and cellular processes (e.g., nuclear DNA damage, mitochondrial activity, and ROS generation); however, the underlying regulatory mechanisms involved in this model remain to be elucidated.

## STAR★Methods

### Key resources table

**Table:** 

REAGENT or RESOURCE	SOURCE	IDENTIFIER
**Bacterial and virus strains**		

*Escherichia coli* DH5a	Takara	Cat#9057

**Chemicals, peptides, and recombinant proteins**		

Tryptone	OXOID	Cat#LP0042B
Yeast extract	OXOID	Cat#LP0021B
Molasses	Solarbio	Cat#FA0070
Sorbitol	Solarbio	Cat#S8090
NaCl	Solarbio	Cat#S8210
SDS	Solarbio	Cat#S8010
Congo red	Solarbio	Cat#C8450
Menadione	Sigma	Cat#M5750-25G
H-89	MCE	Cat#HY-15979
Rapamycin	MCE	Cat#HY-10219
Sucrose	Solarbio	Cat#S8271
Calcofluor white (CFW)	Sigma	Cat#910090
4ʹ,6-diamidino-2-phenylindole (DAPI)	Sigma	Cat#D9524
Fluorescein diacetate (FDA)	Sigma	Cat#F7378
Nile Red	Sigma	Cat#N3013
Propidium iodide (PI)	Sigma	Cat#P4170
Dihydroethidium (DHE)	Beyotime	Cat#S0063
Monodansylcadaverine (MDC)	Sigma	Cat#30432
MitoTracker Red CMXRos	Beyotime	Cat#C1049B
MitoTracker Green	Beyotime	Cat#C1048
Tetraethylbenzimidazolyl-carbocyanine iodide (JC-1)	Beyotime	Cat#C2005

**Critical commercial assays**		

Direct cAMP ELISA kit	Enzo Life Sciences	Cat#ADI-901-066
One-step TUNEL apoptosis detection kit	Beyotime	Cat#C1086
PrimeScriptH^RT^ reagent kit	Takara	Cat#RR037A
LightCycler 480 SYBR Green I Master	Roche	Cat#4887352001

**Experimental models: Organisms/strains**		

*Arthrobotrys oligospora*	ATCC	ATCC: 24927
Δ*Aoras2* mutant strains generated in this study	This paper	NA
Δ*Aoras*3 mutant strains generated in this study	This paper	NA
Δ*Aorheb* mutant strains generated in this study	This paper	NA
*Caenorhabditis elegans*	CGMCC	N2

**Sequence data**		

Transcriptomic data	This paper	PRJNA693160
**Oligonucleotides**		
For all oligonucleotides used for gene manipulation and RT-qPCR	See Table S1	NA

**Software and algorithms**		

pI/MW tool	Expasy	http://web.expasy.org/compute_pi/
InterProScan	Mitchell et al. (2015)	http://www.ebi.ac.uk/Tools/pfa/iprscan/
DNAMAN	Lynnon Biosoft	https://www.lynnon.com/qa.html
MEGA 6	Tamura et al. (2013)	https://www.megasoftware.net/
ImageJ	Schneider et al. (2012)	https://imagej.net/Welcome
Prism 5	GraphPad Software	https://www.graphpad.com/

### Resource available

#### Lead contact

Further information and requests for resources and reagent should be directed to and will be fulfilled by the Lead Contact, Jinkui Yang (jinkui960@ynu.edu.cn).

#### Material availability

This study did not generate new unique reagents.

#### Data and code availability

All data and methods necessary to reproduce this study are included in the manuscript and Supplemental Information. All transcriptomic data are reported in supplemental files of this paper. Sequencing data were deposited to the National Center for Biotechnology Information under accession number PRJNA693160.

### Experimental model and subject details

#### Fungal strains

The WT strain *Arthrobotrys oligospora* Fres. (ATCC24927) and its derived mutants were cultured on PDA plates containing 200 g/L potato, 20 g/L dextrose, and 20 g/L agar at 28°C. For DNA and RNA isolation, mycelium was grown in 500-mL Erlenmeyer flask (EF) containing 250 mL of TG medium (10 g/L tryptone, 10 g/L glucose, and 20 g/L agar) or PDB medium (200 g/L potato and 20 g/L dextrose) and incubated at 28°C and 180 rpm for 3–7 days.

#### Bacterial strain

*Escherichia coli* DH5α was used for cloning of constructs by culturing them in Luria–Bertani broth supplemented with appropriate antibiotics at 37°C.

#### Nematode

The nematode *Caenorhabditis elegans* was incubated at 26°C on oatmeal agar medium. The cultured nematodes were separated from the culture medium using the Baermann funnel technique (Gray, 1984).

### Method details

#### Sequence and phylogenetic analyses of AoRas2, AoRas3, and AoRheb

The amino acid sequences of AoRas2 (GenBank: AOL_s00215g7), AoRas3 (GenBank: AOL_s00083g472), and AoRheb (GenBank: AOL_s00215g525) in *A. oligospora* were downloaded from GenBank (http://www.ncbi.nlm.nih.gov/genbank/). The theoretical isoelectric point (pI) and molecular weight of AoRas2, AoRas3, and AoRheb were calculated using the pI/MW tool (http://web.expasy.org/compute_pi/). Subsequently, their conserved functional domains were analyzed using InterProScan (http://www.ebi.ac.uk/Tools/pfa/iprscan/) with default parameter settings (Mitchell et al., 2015). The orthologous proteins of GTPases AoRas2, AoRas3, and AoRheb were retrieved from various filamentous fungi and their amino acid sequences were downloaded from GenBank. The amino acid sequences of Ras GTPases from different fungi were aligned using the DNAMAN software package (Version 5.2.2, Lynnon Biosoft, St. Louis, Canada). A neighbor-joining tree was constructed using the MEGA 6 software package (Tamura et al., 2013).

#### *Knockout of Aoras2, Aoras3, and Aorheb* genes

Fungal genomic DNA was isolated from 3-day-old cultures of *A. oligospora* mycelia cultured in liquid TG medium. For gene deletion, a knockout cassette was constructed using the homologous recombination approach, as described previously (Colot et al., 2006). The primers used to amplify the flanking sequences for each gene are listed in Table S1. *A. oligospora* genomic DNA was used as a template. Each primer (5r and 3f) has 50 bp tails homologous to the hygromycin-resistance (*hph*) gene cassette. The *hph* was amplified using the primers hphF and hphR and the plasmid pCSN44 as a template. Subsequently, the three DNA fragments (5ʹ-flanking region, *hph* cassette, and 3ʹ-flanking region) and a pRS426 backbone (digested by *Eco*RI and *Xho*I) were used to transform the yeast *Saccharomyces cerevisiae* strain FY834 through electroporation (Christianson et al., 1992). Circular constructs were synthesized using homologous recombination, followed by the extraction of total transformant DNA. The final disruption vectors (pRS426–target gene–hph) were extracted after transformation into *E. coli* DH5α. Thereafter, the plasmids were extracted and the recombination was confirmed using PCR with the primers 5f and 3r (Table S1) for each gene. The whole knockout cassette containing the *hph* gene with two homologous recombination arms was amplified through PCR with the primers 5f and 3r and was then used to transform into *A. oligospora* by following a protoplast-based protocol (Tunlid et al., 1999). Transformants were selected on PDAS (PDA supplemented with 0.6 M sucrose) containing 200 μg/mL hygromycin B. The positive transformants were further confirmed through PCR amplification using the primers Yf and Yr for each gene (Table S1) and Southern blot analyses. Southern blot analysis was carried out using the North2South chemiluminescent hybridization and detection kit (Pierce, Rockford, USA), following the manufacturer's instructions. The primer pairs Tf and Tr of each gene (Table S1) were used to generate the Southern hybridization probe using PCR. The restriction enzymes *Xho*I, *Sac*I, and *Hin*dIII, were used respectively to digest the genomic DNA of *A. oligospora* and corresponding mutants (Δ*Aoras2*, Δ*Aoras3*, and Δ*Aorheb*) for Southern blot analysis.

#### Comparison of mycelia growth and morphology between the WT and mutants

The mycelial plugs of fungal strains were inoculated on PDA, TYGA (10 g/L tryptone, 5 g/L yeast extract, 10 g/L glucose, 5 g/L molasses, and 20 g/L agar), and TG medium and incubated at 28°C for 7 days each, and their growth rates and colony morphologies were observed. The experiment was repeated three times for each strain.

#### Comparison of stress resistance

The mycelial plugs of each strain were inoculated into TG media supplemented with different concentrations of chemical stressors at 28°C for 5–7 days. The chemical stressors employed for the experiment were as follows: sorbitol (0.25, 0.5, and 1 M) and NaCl (0.1, 0.2, and 0.3 M) as osmotic stressors, SDS (0.01–0.03%) and Congo red (20–100 ng/mL) as CWPAs, and H_2_O_2_ (5–15 mM) and menadione (0.01–0.05 mM) as oxidative stressors. H-89 (100 mM/mL) and rapamycin (5–15 ng/mL) were used as the inhibitors of PKA and TOR signaling, respectively. The RGI values of every colony were calculated as previously described (Zhen et al., 2018).

The growth rates of WT strain and mutants were compared with determine their recuperation, following heat shock, after inoculating them on PDA plates for 2 days at 28°C and for 8 hr at 28, 34, 38, or 40°C, followed by incubation at 28°C for 7 days, after which the colony diameter was determined as previously described (Zhen et al., 2019).

#### Comparison of conidial yield and germination

The mycelial plugs of each mutant and WT strain that were of the same size and age were inoculated in 250-mL EF supplemented with 30 mL of cornmeal-molasses-yeast (CMY) agar medium (20 g/L maizena, 20 g/L agar, and 5 g/L yeast extract), followed by incubation at 28°C for 15 days. The conidia of WT and mutants were collected from CMY medium by scraping them off with a glass spatula into 10 mL sterile distilled H_2_O, followed by filtration through four layers of lens tissues to remove mycelial debris. The conidia were washed with 10 mL water, followed by centrifugation (2 min, 12,000 rpm) to remove the liquid from the conidial pellets each time and resuspension in 1 mL sterile distilled H_2_O. The number of microscopic counts of conidia was determined using a hemocytometer (Doehlemann et al., 2006).

For germination tests, following the method previously described (Garcia-Rico et al., 2011) with minor modifications, the conidia of each mutant and WT strain were obtained from CMY medium through filtration, centrifugation (2 min, 12,000 rpm), and suspension in 1 mL sterile distilled H_2_O to achieve a final concentration of conidia of 10^5^ spores/mL. This 1 mL of sterile distilled H_2_O containing conidia was then inoculated in 30 mL Vogel's medium (minimal) (20 mL/L Vogel's salts and 15 g/L sucrose) in a 250-mL EF and incubated at 28°C and 180 rpm. At regular intervals of 4 hr, 1-mL samples were removed, and triplicates of 20 μL were observed under the microscope to count the number of germinated and non-germinated conidia. The experiments were performed in triplicate.

#### Bioassay against the nematode C. elegans

Approximately 1 × 10^4^ conidia were cultivated on WA plates. After 3 days of incubation at 28°C, 200 nematodes (*C. elegans*) were added to the center of each plate. The number of traps and captured nematodes were counted under a light microscope (BX51, Olympus, Tokyo, Japan) after 12, 24, 36, and 48 hr.

#### Determination of proteolytic activity

The fungal strains were inoculated in PDB medium and incubated at 28°C and 180 rpm for 7 days. The fermentation liquid was collected and the protease activity was determined on casein plates (Zhao et al., 2004). Moreover, the protease activity was determined using a caseinolytic method as described previously (Wang et al., 2006). One unit (U) of protease activity was defined as the amount of enzyme that hydrolyzed the substrate and produced 1 μg of tyrosine in 1 min under specific assay conditions.

#### Quantification of intracellular cAMP levels

Intracellular cAMP levels were determined as described previously (Liu et al., 2007). Fungal strains were inoculated on PDB medium and incubated at 28°C. The mycelia were then harvested on 3, 5, and 7 days, treated with 1 M HCl for 30 min, and stored in liquid nitrogen. Intracellular cAMP was extracted and their levels were quantified using a direct cAMP ELISA kit (Enzo Life Sciences, New York, USA) according to the manufacturer's protocol.

#### Transcriptome sequencing and analysis

The WT and ΔAoras2 mutant strains were cultured on PDA plates for 3 days and the mycelia of fungal strains were treated with *C. elegans* from 0 to 48 hr, and 30 samples were obtained for further analysis. Mycelial samples were sent to Wuhan Seqhealth Technology Co. Ltd. for transcriptome sequencing, and the data were analyzed using KOBAS 3.0 (http://kobas.cbi.pku.edu.cn/kobas3/?t=1). Reads per kilobase per million mapped reads (RPKM) of all DEGs with annotation. The DEGs in the ΔAoras2 mutant at each time point were analyzed using STRING (https://string-db.org/), followed by further visualization and analysis with Cytoscape (https://cytoscape.org/). After the analysis, several genes were amplified using reverse transcription qualitative PCR (RT-qPCR) to verify the transcriptome data obtained (Zhang et al., 2019a) (Table S9). The genes and their primers are listed in the Table S1.

#### HPLC-MS and untargeted metabolomic analyses

The WT and Δ*Aoras2* and Δ*Aorheb* mutant strains were inoculated in 500-mL EF containing 250 mL of PDB medium and incubated at 28°C and 180 rpm for 7 days. After the incubation, the cultures were filtered to separate the fermentation liquid from the mycelia and the wet weight of the mycelia for each strain was measured. The wet weights of mycelia from WT and the mutant strains (Δ*Aoras2* and Δ*Aorheb*) were determined, and the volume of the fermentation broth corresponding to the equal biomass (wet weight) of WT and mutant strains was added to the culture. The culture filtrate was extracted using ethyl acetate (1:1 v/v) and the extraction was performed for 12 hr. The ethyl acetate fractions were concentrated *in vacuo* to yield residues, and the dried organic residues were then dissolved in methanol. The solution was filtered through a 0.22-μm membrane filter for further analysis with HPLC-MS.

An ultrahigh-performance LC system combined with a Q exactive focus orbitrap mass spectrometry (Thermo Fisher Scientific, Bremen, Germany) equipped with electrospray ionization mode and an Agilent Zorbax ODS 4.6 × 250 mm column (Agilent, Santa Clara, CA, USA) was used for sample analysis. Column temperature was fixed at 40°C and the injection volume was 10 μL. Full scan mode with a scan range from *m*/*z* 100 to 1000 was specified for orbitrap mass analyzer. UV spectra were recorded at 220 to 400 nm. Mobile phase A was 0.1% formic acid in water and mobile phase B was 0.1% formic acid in acetonitrile. The LC conditions were specified as described previously and were further manually optimized based on separation patterns as follows: gradient program of B (0 min 10% B; 2 min 10% B; 10 min 25% B; 30 min 35% B; 35 min 50% B; 45 min 90% B; 47 min 10% B; 49 min 10% B) (Chen et al., 2020; He et al., 2019). The flow rate was adjusted to 1 mL/min with a linear gradient program. Three replicates were maintained for the analysis. Untargeted metabolomics analysis was performed using the Compound Discoverer 3.0 software (Thermo Fisher Scientific, CA, USA).

#### Morphological observation, nuclear staining, and TUNEL analysis

For the observation of mycelial and conidial morphology, the WT and mutant strains were stained with 10 μg/mL calcofluor white stain (Sigma-Aldrich, St. Louis, MO, USA), and the cell nuclei of mycelia and conidia were visualized by staining the cells with 10 μg/mL DAPI for 30 min (Guo et al., 2011). The samples were then washed with distilled water and observed under a confocal laser scanning microscope (Leica, Mannheim, Germany) or light microscopy.

DNA fragmentation and cell apoptosis were determined via the TUNEL assay, followed by staining the nucleus with 10 μg/mL PI for 10 min. TUNEL assay using the one-step TUNEL apoptosis detection kit (Beyotime, Jiangsu, China) was performed according to the manufacturer's protocol. The cells were then observed under a confocal laser scanning microscope and the fluorescence intensity was estimated using the ImageJ software (https://imagej.net/Welcome) (Schneider et al., 2012).

#### Analysis of the spore activity, ROS level, LD formation, and autophagic activity

Spore activity was observed by staining the cells with 10 μg/mL FDA for 10 min, followed by washing them with distilled water. ROS, LDs, and autophagosomes were stained with 10 μg/mL DHE, Nile Red, and MDC, respectively, for 10 min. The samples were then washed with distilled water and observed under a confocal laser scanning microscope. The fluorescence intensity was determined using the ImageJ software (https://imagej.net/Welcome) (Schneider et al., 2012).

#### Mitochondrial morphology and mitochondrial membrane potential analysis

The morphology of mitochondria was observed by staining the cells with 10 μg/mL MitoTracker Red CMXRos (Beyotime, Jiangsu, China) or MitoTracker Green (Beyotime, Jiangsu, China), in which the MitoTracker Red CMXRos could specifically label the bioactive mitochondria in cells. The number of mitochondria was determined using a confocal laser scanning microscope.

Mitochondrial membrane potential was determined by staining the cells with 10 μg/mL JC-1 (Beyotime, Jiangsu, China) for 10 min and then washing them with distilled water. The cells were observed under a confocal laser scanning microscope and the fluorescence intensity was determined using the ImageJ software.

#### RT-qPCR analysis

Total RNA was isolated from the samples using an RNA extraction kit (Axygen, Jiangsu, China) and reverse transcribed into cDNAs with a PrimeScriptH^RT^ reagent kit (with gDNA, TaKaRa, Kusatsu, Japan). The cDNA was used as a template to determine the mRNA expression of candidate genes associated with phenotypes, such as conidiation and stress resistance, by performing RT-qPCR with specific paired primers and the LightCycler 480 SYBR Green I Master (Roche, Basel, Switzerland) (Table S1). β-tubulin was used as an internal standard. All RT-qPCR experiments were performed in triplicates. Relative transcript level (RTL) of each gene was computed as the ratio of cycle threshold values for the gene in the mutant strain to that in the WT strain using the 2^−ΔΔCt^ method (Livak and Schmittgen, 2001).

### Quantification and statistical analysis

Each experiment was performed in triplicate, and the data from each experiment were expressed as mean ± standard deviation (SD). One-way analysis of variance (ANOVA) followed by honestly significant difference (HSD) test were used for statistical analyses. p values <0.05 were considered significant (Farnesi et al., 2015). All statistical analyses were performed using the GraphPad Prism software version 5.00 for Windows (GraphPad Software, San Diego, California, USA).

## Supplemental information

Document S1. Figures S1–S6

Table S1. List of primers used in gene manipulation and reverse transcription qualitative PCR (RT-qPCR), Related to STAR Methods

Table S2. Sequencing data statistics of transcriptome, Related to Figures 6 and S4

Table S3. Lists of Gene Ontology (GO) enrichment of differentially expressed genes, Related to Figure 6

Table S4. The genes in Gene Ontology (GO) enriched at each time point may be related to trap formation and pathogenicity, Related to Figure 6

Table S5. Kyoto Encyclopedia of Genes and Genomes (KEGG) pathway enrichment of differentially expressed genes at different time points, Related to Figure 6

Table S6. The protein–protein interaction network of differentially expressed genes (DEGs) in the ΔAoras2 mutant at each time point was analyzed via STRING, Related to Figure 6

Table S7. Annotation of the metabolites present in the ΔAoras2 and ΔAorheb mutants, Related to Figure 9

Table S8. Kyoto Encyclopedia of Genes and Genomes (KEGG) enrichment of differentially expressed metabolites, Related to Figure 9

Table S9. The transcriptome was analyzed for the presence of 46 differentially expressed genes (DEGs) using reverse transcription qualitative PCR (RT-qPCR) at 12 h, Related to Figure 10
