# Supplementary material for: Pleiotropic roles of Ras GTPases in the nematode-trapping fungus Arthrobotrys oligospora identified through multi-omics analyses
Source: iScience. 2021 Jul 8;24(8):102820. doi: 10.1016/j.isci.2021.102820 (PMC8313493; doi:10.1016/j.isci.2021.102820)
Supplement: Document S1. Figures S1–S6 [file mmc1.pdf]

iScience, Volume *24*

Supplemental information

Pleiotropic roles of Ras GTPases in the nematode-trapping fungus *Arthrobotrys oligospora* identified through multi-omics analyses

Le Yang, Xuemei Li, Meihua Xie, Na Bai, Jiangliu Yang, Kexin Jiang, Ke-Qin Zhang, and Jinkui Yang

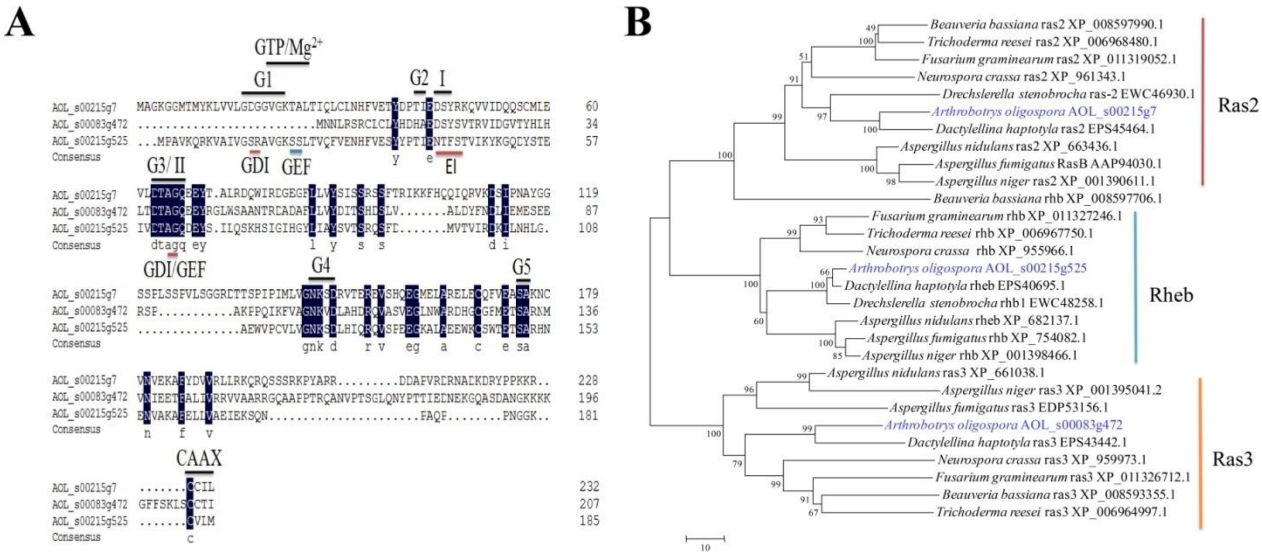

Figure S1 Multiple sequence alignment and phylogenetic analyses of Ras GTPases. Related to Table 1.

(A) The conserved functional domains were analyzed using InterProScan 51.0 (http://www.ebi.ac.uk/Tools/pfa/iprscan/). Areas shaded in black suggest conserved regions (100% similarity). Several conserved domains are marked, such as G1 to G5 box and CAAX. (B) Phylogenetic relationship among orthologous Ras2, Ras3, and Rheb proteins from *A. oligospora* and other fungi. The orthologs of Ras2 (AoRas2), Ras3 (AoRas3), and Rheb (AoRheb) in *A. oligospora* are marked in blue color.

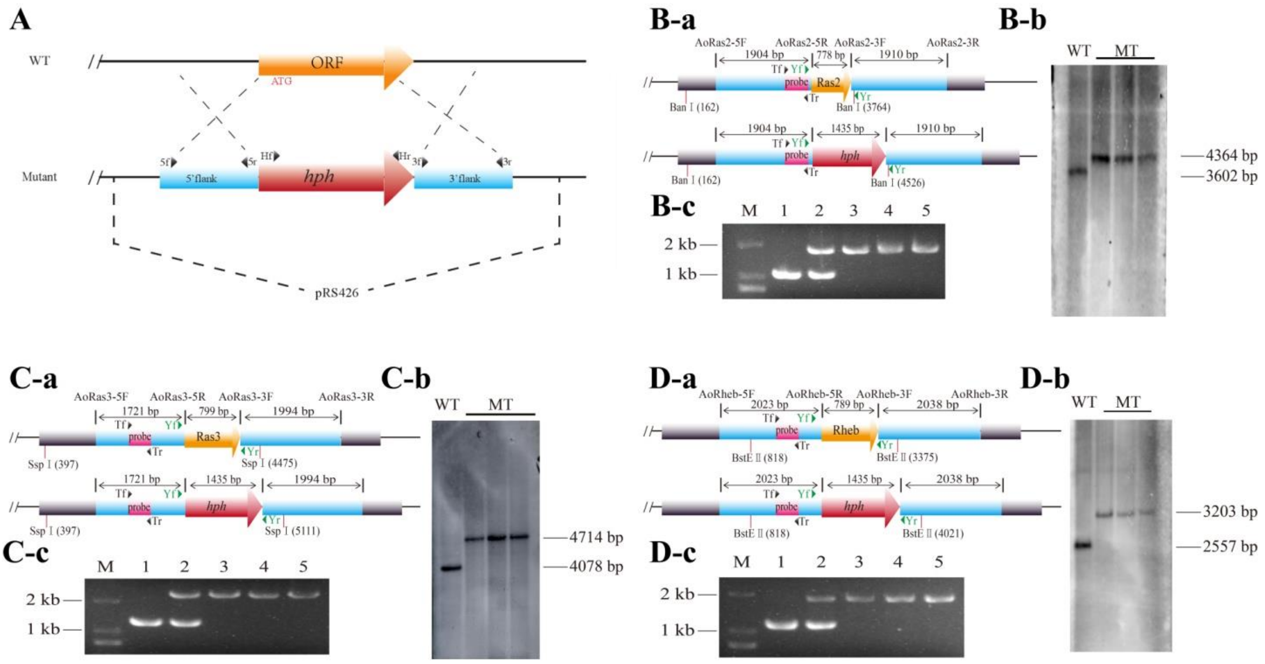

Figure S2 Verification of the knockdown of Ras GTPase genes in *A. oligospora*. Related to Figure 1.

(A) Diagrammatic representation of homologous recombination of Ras GTPase genes in *A. oligospora*. (B) Verification of the *Aoras2* gene knockout using PCR and Southern blot analyses. a. The diagrammatic representation of homologous recombination of the *Aoras2* gene. Primers AoRas2-5f/AoRas2-5r and AoRas2-3f/AoRas2-3r were used for the amplification of homologous flanks of the target gene, and the primers AoRas2-Yf/AoRas2-Yr (Supplementary Table S1) were used for the verification of transformants. *Probe* indicates the site of the Southern blot probe, and *Ban*I was the restriction enzyme used for Southern blot analysis. b. The transformants of gene *Aoras2* were verified using PCR with the primers AoRas2-Yf/AoRas2-Yr. Line 3, 4, and 5 suggest positive transformants, line 1 suggests the wild-type (WT) strain, whereas line 2 suggests heterozygotic transformant with a WT gene copy and *hph*-replaced copy. M represents the DNA marker. c. Southern blot analysis of the WT and Δ*Aoras2* mutants. MT represents three independent mutants. (C) Verification of the *Aoras3* gene knockout using PCR and Southern blot analysis. a. The diagrammatic representation of homologous recombination of the *Aoras3* gene. Primers AoRas3-5f/AoRas3-5r and AoRas3-3f/AoRas3-3r were used for the amplification of homologous flanks of the target gene, and the primers AoRas3-Yf/AoRas3-Yr (Supplementary Table S1) were used for the verification of transformants. *Probe* indicates the site of the Southern blot probe, and *Ssp*I was the restriction enzyme used for Southern blot analysis. b.

The transformants of gene *Aoras3* were verified using PCR with the primers AoRas3-Yf/AoRas3-Yr. Line 3, 4, and 5 suggest positive transformants, line 1 suggests the wild-type (WT) strain, whereas line 2 suggests heterozygotic transformant with a WT gene copy and *hph*-replaced copy. M represents the DNA marker. c. Southern blot analysis of the WT and Δ*Aoras3* mutants. MT represents three independent mutants. (D) Verification of the *AorheB* gene knockout using PCR and Southern blot analysis. a. The diagrammatic representation of homologous recombination of the *AorheB* gene. Primers AoRheB-5f/AoRheB-5r and AoRheB-3f/ AoRheB-3r were used for the amplification of homologous flanks of the target gene, and the primers AoRheB-Yf/AoRheB-Yr (Supplementary Table S1) were used for the verification of transformants. *Probe* indicates the site of the Southern blot probe, and *Bst*EII was the restriction enzyme used for Southern blot analysis. b. The transformants of gene *AorheB* were verified using PCR with the primers AoRheB-Yf/AoRheB-Yr. Line 3, 4, and 5 suggest positive transformants, line 1 suggests the wild-type (WT) strain, whereas line 2 suggests heterozygotic transformant with a WT gene copy and *hph*-replaced copy. M represents the DNA marker. c. Southern blot analysis of the WT and Δ*AorheB* mutants. MT represents three independent mutants.

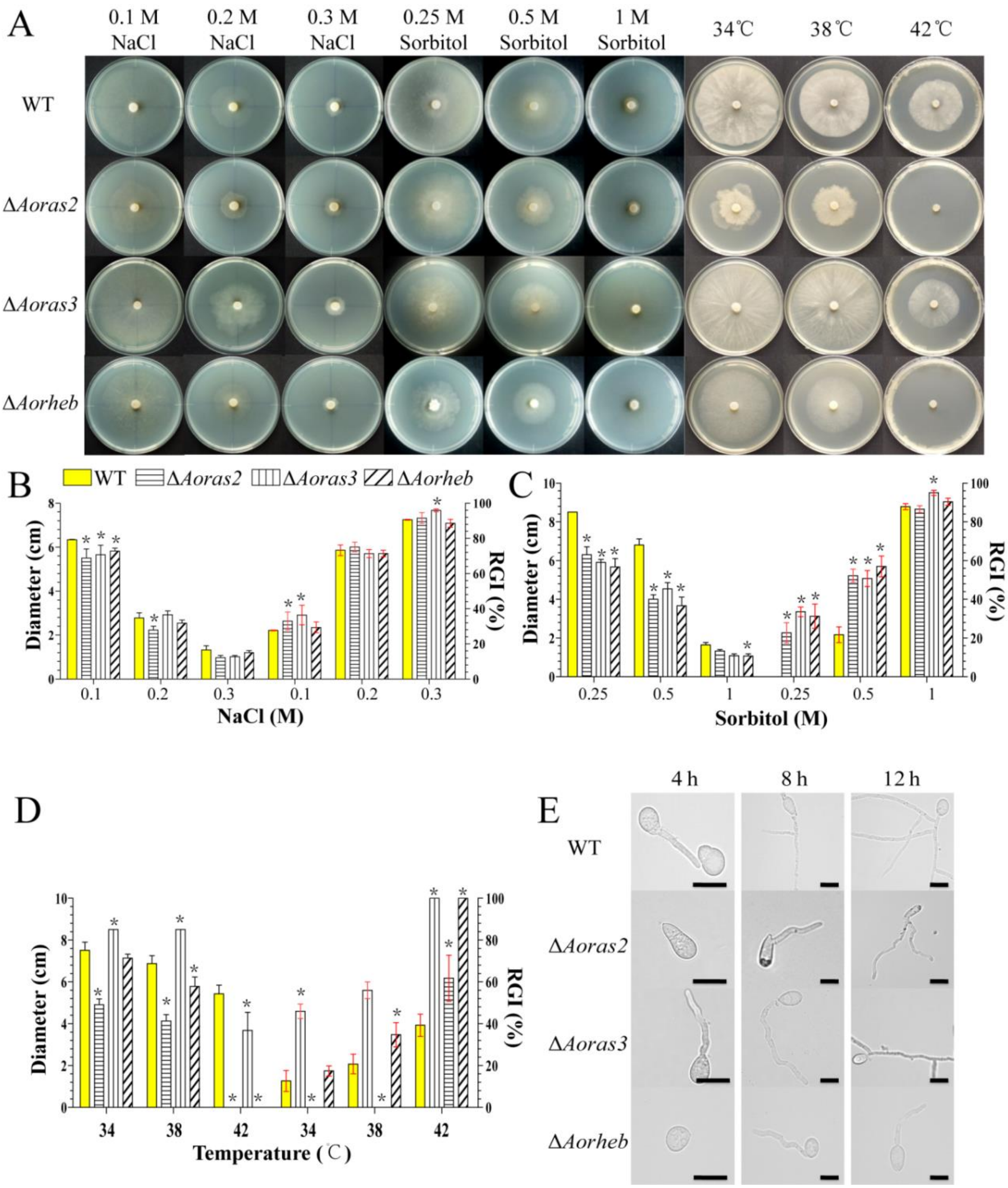

Figure S3 Comparison of stress tolerance to osmotic agents, heat shock, and conidial germination. Related to Figure 3.

(A) Colony morphologies of the WT and mutant strains under stress conditions such as osmotic agents and heat shock. (B–D) Colony diameters and the relative growth inhibition (RGI) values of the strains cultured in the presence of (B) 0.10–0.30 M NaCl, (C) 0.25–1 M sorbitol, (D) heat shock (34, 38, and 42 °C). Error bars: Data are represented as mean ± SD. An asterisk in (B–D) indicates a significant difference between mutants and the WT strain (n = 3 for the WT strain, n = 9 for the each mutant strain; Tukey's HSD, $P < 0.05$). (E) Spore germination of the WT and mutant strains at different time points. Bar = 20 μm.

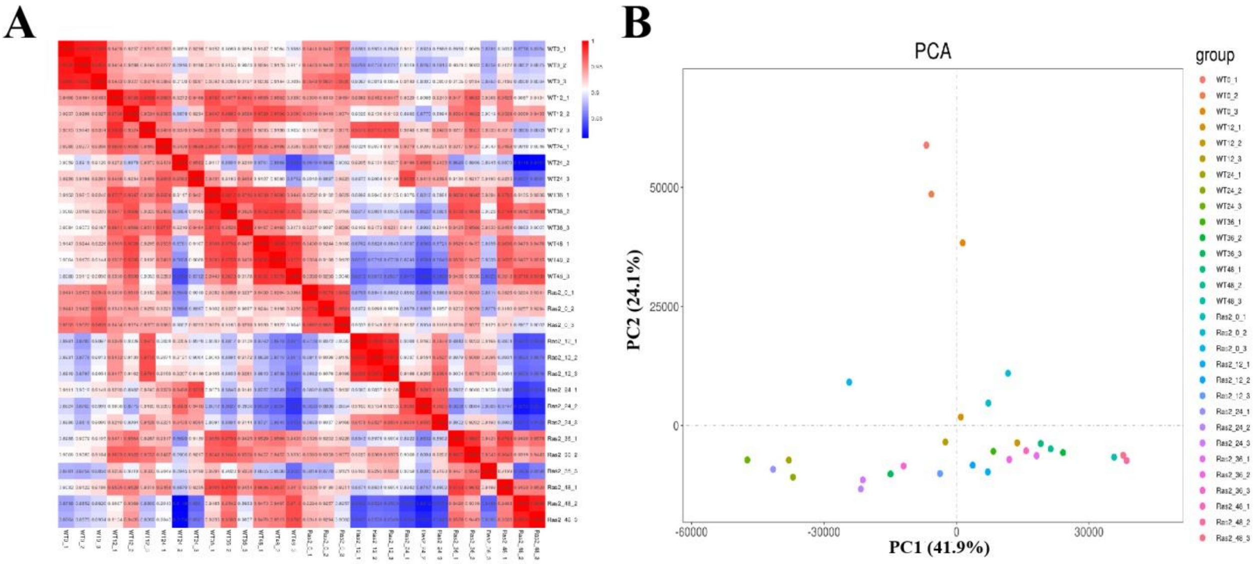

Figure S4 Determination of the Spearman correlation coefficient and principal component analyses (PCA) for each sample. Related to Figure 6.

(A) The Spearman correlation coefficient estimated for three biological samples at each time point. Spearman correlation coefficient is above 0.943 and 0.942 for the Δ*Aoras2* and WT, respectively. (B) PCA analyses of the WT and Δ*Aoras2* mutant strains were performed on three biological samples at each time point.

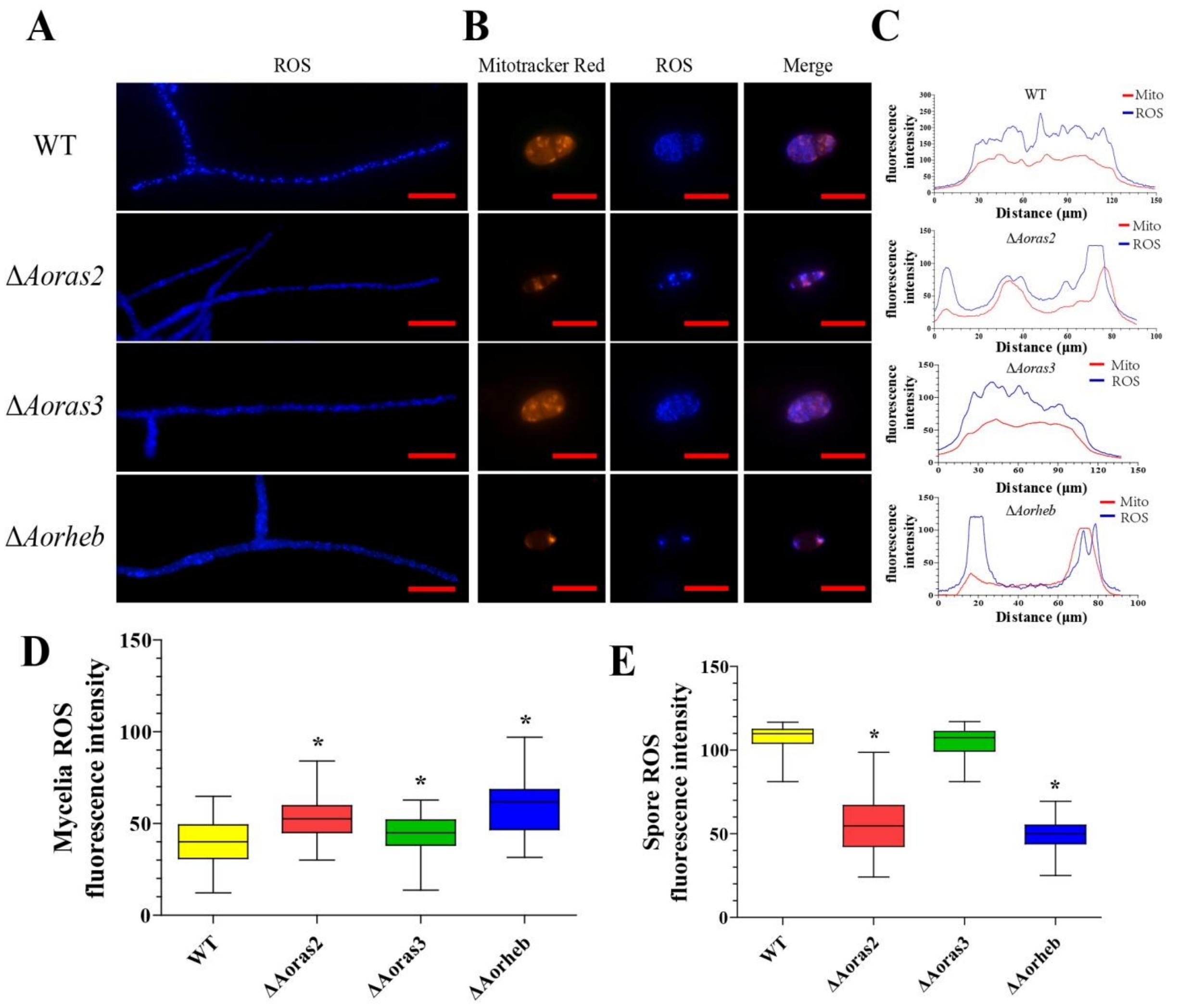

Figure S5 Effect of AoRas2 and AoRheb on ROS levels in mycelia and spores. Related to Figure 10.

(A) Mycelia and spores of the WT and mutants were stained with dihydroethidium (DHE). (B) Spores of the WT and mutants were stained with DHE and MitoTracker Red CMXRos. Samples (A) and (B) were examined under a confocal laser scanning microscope. Bar = 10 μm. (C) The distribution of ROS and mitochondria in spores was observed through the co-localization of dyes using the ImageJ software. (D, E) The ROS content of WT and mutant strains in (D) hyphae and (E) spores was analyzed by estimating DHE fluorescence intensity values for at least 30 fields viewed under a microscope, and the horizontal bars depict the median. An asterisk in (D, E) indicates a significant difference between mutants and the WT strain ($P < 0.05$).

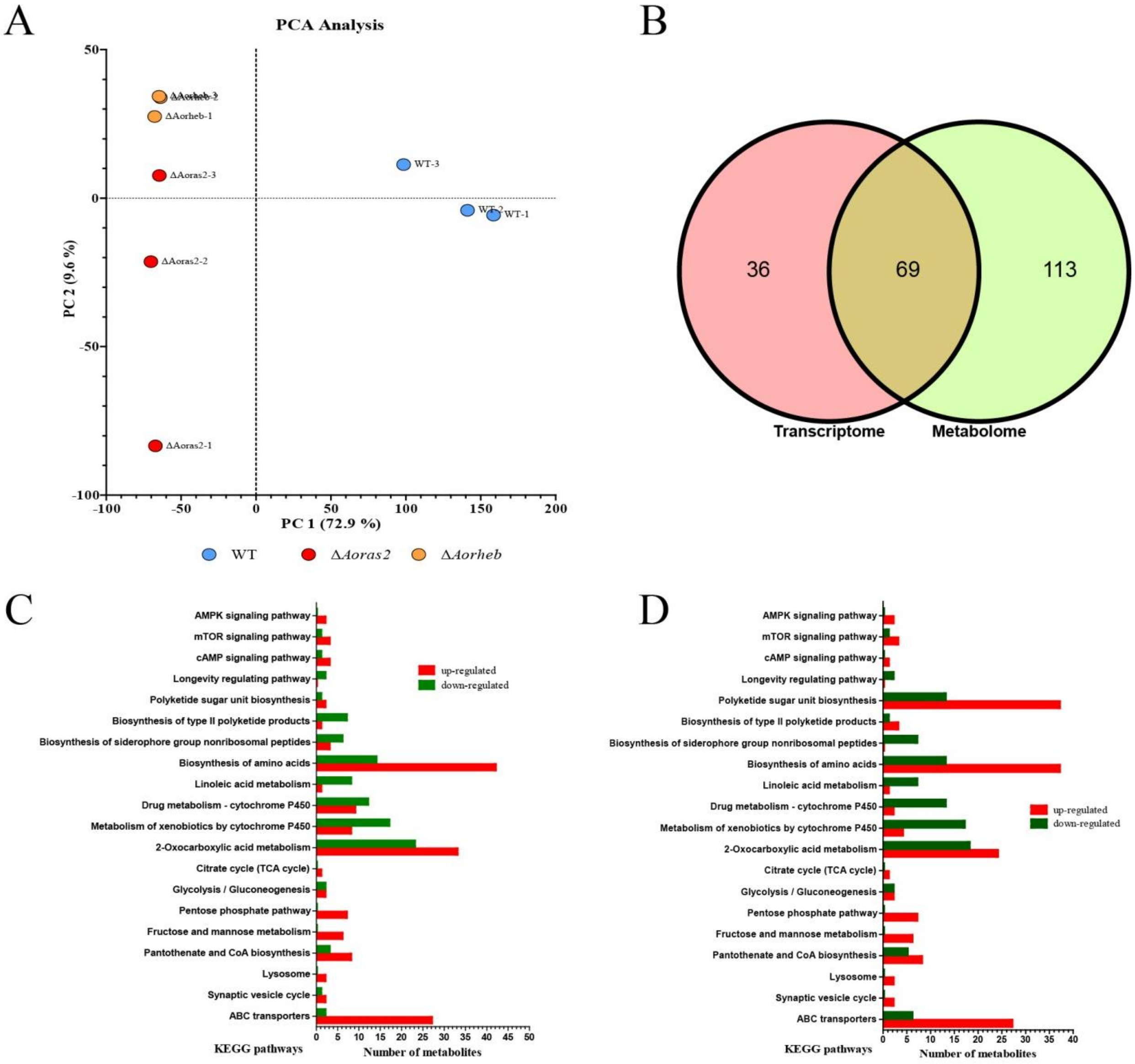

Figure S6 Comparison of untargeted metabolome using principal component analysis and partial KEGG enrichment. Related to Figure 9.

(A) PCA was used to analyze the metabolites of the WT and mutant strains (Δ*Aoras2* and Δ*Aorheb*). Three biological samples were maintained for each strain. (B) Venn analysis of the KEGG pathways in the transcriptome and metabolome. There are 69 KEGG pathways common between transcriptome and metabolome. (C) Partial KEGG pathway enrichment of differentially expressed metabolites (DEMs) in the Δ*Aoras2* mutant strain. (D) Partial KEGG pathway enrichment of DEMs in the Δ*Aorheb* mutant.
